# Integrative in-silico and in-vitro analysis of taurine and vitamin B12 in modulating PPARγ and Wnt signaling in hyperhomocysteinemia-induced osteoporosis

**DOI:** 10.1186/s13062-024-00581-z

**Published:** 2024-12-20

**Authors:** Mazumder Adhish, I. Manjubala

**Affiliations:** School of Bio Sciences and Technology, Vellore Institute of Technology, Vellore, Tamil Nadu India

**Keywords:** PPARγ, Taurine, Homocysteine, Vitamin B12, Osteoporosis, Hyperhomocysteinemia, Molecular docking, Molecular dynamics, Wnt signaling

## Abstract

Peroxisome proliferator-activated receptor-γ (PPARγ) is a critical regulator of adipogenesis and bone metabolism, playing complex roles in osteoporosis. This study investigates the effects of taurine and homocysteine on PPARγ, focusing on their roles in osteoclastogenesis and bone health. In-silico analyses, including molecular docking and molecular dynamic simulations, revealed that both taurine and homocysteine bind competitively to the PPARγ ligand-binding domain, exhibiting distinctive antagonistic modes, including destabilization of PPARγ’s key helices H3, H4/5, H11, and H12. In-vitro experiments further supported these results, demonstrating that taurine protects against oxidative damage, enhances bone mineralization, and reduces the expression levels of PPARγ, while also downregulating negative regulators of the Wnt signaling pathway, such as SOST and DKK1. Homocysteine, on the other hand, was observed to increase the expression of these regulators and impair bone formation. Vitamin B12 was included in the study due to its known role in mitigating hyperhomocysteinemia, a condition linked to impaired bone health and reduced taurine levels. While vitamin B12 alone demonstrated some beneficial effects, it did not achieve the same level of efficacy as taurine. However, a combination of taurine and vitamin B12 showed greater efficacy in ameliorating hyperhomocysteinemia-induced osteoporosis. Overall, this study highlights taurine’s therapeutic potential in counteracting the adverse effects of hyperhomocysteinemia on bone health and underscores the need for further research into taurine’s mechanisms in osteoporosis treatment.

**Graphical abstract:**

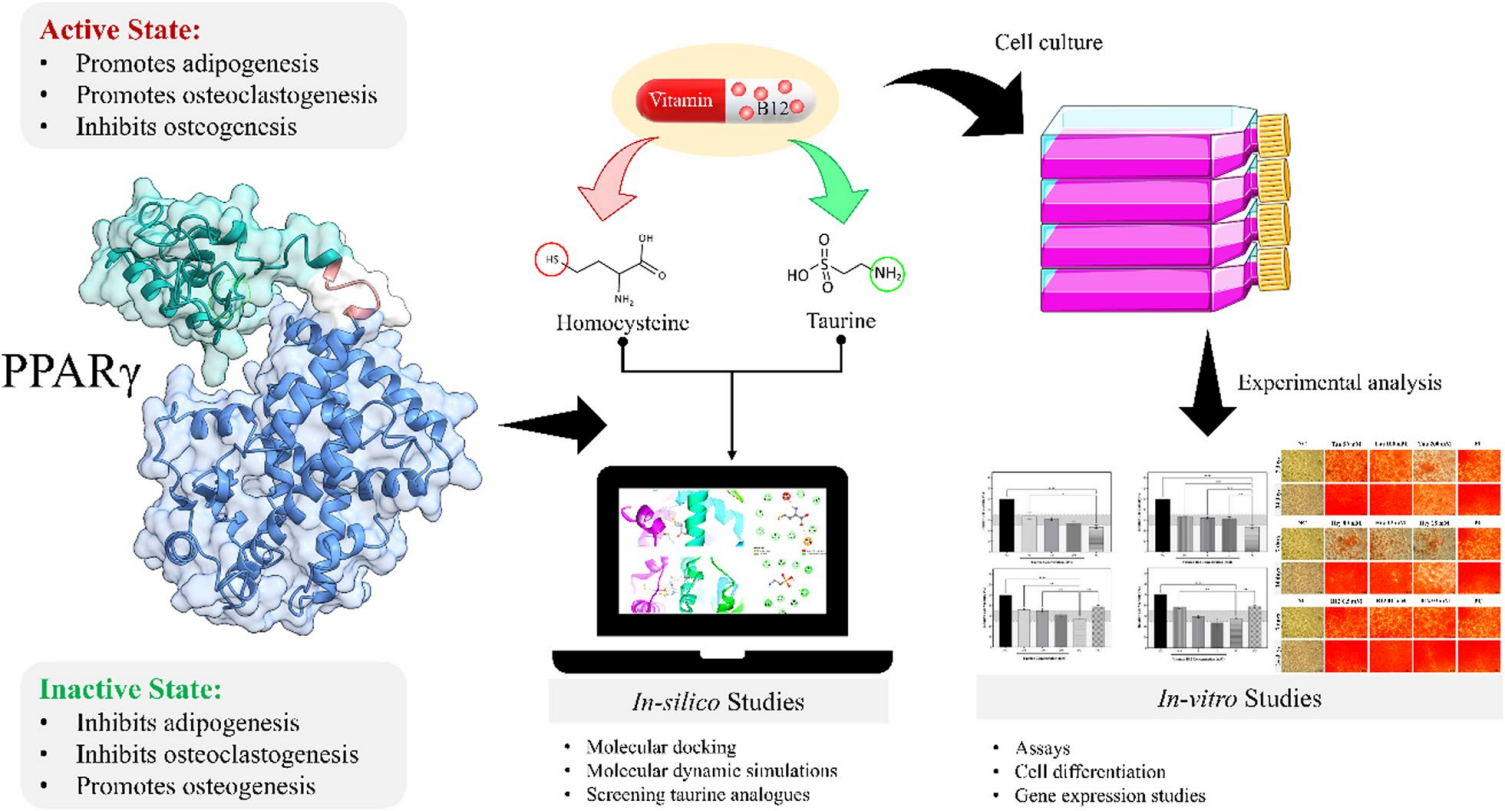

**Supplementary Information:**

The online version contains supplementary material available at 10.1186/s13062-024-00581-z.

## Introduction

Osteoporosis is a progressive disorder of the skeletal system characterized by the thinning of the trabecular and the cortical bones in the body leading to a decrease in bone mass and reduction in the formation of bone tissues around the bone causing damage to the trabecular system prevalent in bones. This makes the bones more susceptible to getting fractured easily owing to the fact that the bone strength gets compromised as bone resorption by the osteoclasts exceeds bone formation by the osteoblasts, resulting in the net loss of the entire bone integrity [1, 2]. Hyperhomocysteinemia on the other hand demonstrates an absurd increase in the levels of systemic homocysteine which ultimately leads to a rise in toxic conditions in the body. Over the years, a causal relationship between hyperhomocysteinemia (HHcy) and osteoporosis has been established [3]. Homocysteine is basically a sulfhydryl- containing amino acid formed as an intermediary product of methionine metabolism, whose role in degradation of collagen through its interference in the formation of collagen cross-links [3], estrogen receptor hypermethylation and thereby its subsequent downregulation [4] and NF-κΒ mediated increase in oxidizing environment [5] has already been studied. Even though homocysteine’s role in the promotion of osteoclastogenesis through the introduction of oxidative stress has been identified, the mechanism of its action has remained speculative over the years with studies associating it with the anti-N-methyl-D-aspartate receptor (NMDA-R) as a ligand resulting in an increase in the overall reactive oxygen species (ROS) levels within the cell through an influx of calcium and inadvertently an oxidizing environment [6]. In addition to that, its role upon binding to PPARγ as a ligand remains contradicting in literature and not confirmed [7–9].

**Fig. 1:**
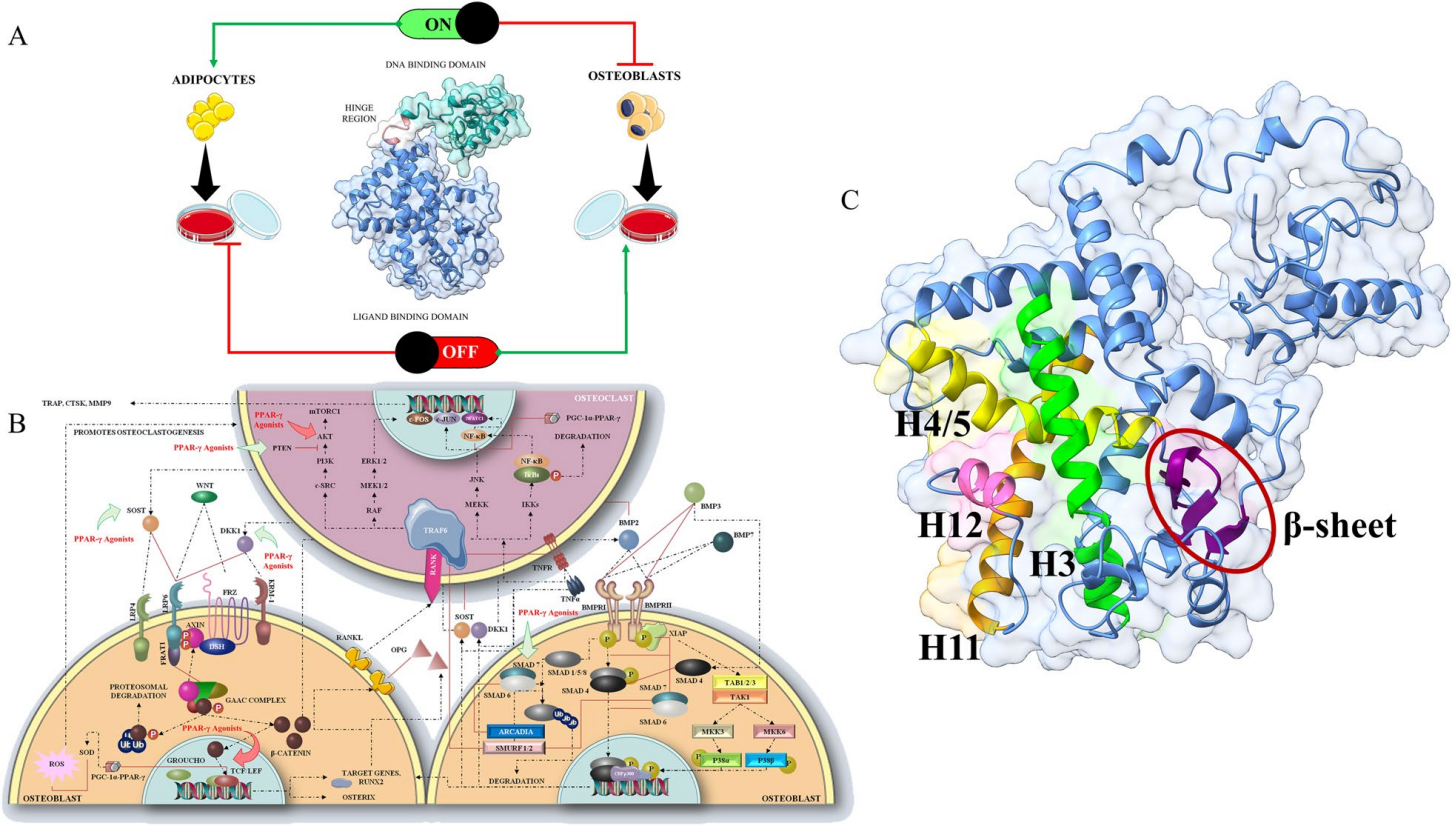
The figure demonstrates PPARγ and its relation to signaling pathways. The panel **A** shows PPARγ as the molecular switch between adipogenesis and osteogenesis; **B** demonstrates the mechanism by which PPARγ agonists target the methodical inhibition of osteoblastogenesis and initiation of osteoclastogenesis; and **C** denotes the important helices H3, H4/5, H11 and H12 targeted by agonists and antagonists of PPARγ and in addition to them the β-sheets which are targeted by partial agonists and inverse agonists

Peroxisome proliferator-activated receptor—γ (PPARγ) belongs to a nuclear receptor family of transcription factors that depend on their ligands for its proper expression and the regulation of several functions such as adipogenesis, homeostasis of glucose, homeostasis of energy, antioxidant properties, inflammation, autophagy and bone metabolism [10–14]. Like most of the other nuclear receptors, PPARγ has a N-terminal region called activation function-1 (AF-1) which is usually disordered functioning as a binder of co-regulators, a zinc-finger domain which acts as the region which binds with the DNA (DBD), a domain for binding with ligands (LBD) which includes the activation function-2 (AF-2) region which is necessary for the proper binding with ligands or co-activators and finally a hinge region between LBD and DBD which holds the two domains together but is extremely mobile. Its structure comprises of 13 α-helices and a β-sheet with 4 strands [15] with the AF-2 region known to bind with co-activators being one of the most crucial regions for determining whether PPARγ would be activated or repressed. Even though a lot is known about the way agonists interact with PPARγ to elicit its role in various processes—predominantly adipogenesis, PPARγ antagonism is just starting to be understood in relation to its role in bone biology and especially in chronic bone diseases like osteoporosis. It is understood thus far that the PPARγ agonists mostly rely on the stabilization of helix 12 (H12), helix 11 (H11), helix 4—helix 5 (H4-H5) and helix 3 (H3) [16–18] of PPARγ – thereby giving rise to the AF-2 surface which is necessary for the binding of co-activators (Fig. 1). The β1-4 sheets region is considered important for the transactivation and transrepression of PPARγ through partial agonists and inverse agonists respectively which very rarely stabilizes H12 [16, 19, 20]. Antagonists of PPARγ are therefore presumed to act upon this area consisting of H12, H11, H4-H5 and H3 for destabilizing H12 such that the AF-2 is disturbed and the co-activators do not get recruited for binding to PPARγ and activating them. PPARγ antagonism is connected with the instigation of osteogenesis in place of adipogenesis, thus forming a molecular switch between the two varied processes catering two different systems within the human body. Thus, as indicated by studies performed in the field, the activation of PPARγ promotes osteoclastogenesis and inhibition of osteoblastogenesis through the direct inhibition of Wnt pathway by the downregulation of β-catenin [21]. When Wnt pathway is active, it represses PPARγ both directly through the canonical and indirectly through the non-canonical pathways [22]. This in addition to the fact that PPARγ promotes sclerostin (SOST) production [23] which is considered one of the best targets for osteoporosis [24–27] and the fact that PPARγ is associated with the regulation of ROS, makes it a lucrative target for osteoporosis as well.

Another function of PPARγ which has been brought forth in recent years is the regulation of taurine transporter (TauT) expressions in the body [28]. Taurine transporters are a form of transport system in the body which is responsible for the co-transportation of taurine inside cells and across basolateral membranes along with two sodium ions and one chloride ion, found to be expressed in osteoblasts and osteoclasts as well [29]. Taurine, which is an amino sulfonic acid arising from the same biosynthetic pathway as homocysteine, has been noted to exhibit a completely opposite effect when compared to homocysteine on the body. Even though taurine was initially thought to be biologically inert [30], its functional versatility in the biological domain of humans such as an antioxidant, anti-proliferative, anti-endoplasmic reticulum stress inducer and anti-inflammatory compound [31] while also behaving as an osmoregulatory agent, ion channel regulator, stabilizer of proteins and cell membranes, and regulator of intracellular calcium homeostasis has been well documented by now [32]. Even though the discovery of its myriad functions have caused a resurgence of interest in understanding this compound in several areas of the human health and the resultant documentation of its mechanism of action on such systems, the knowledge about its role in bone biology is still heavily shrouded in mystery. Even though a decrease in taurine levels has been seen in patients of osteoporosis [33], the mechanism of its action on bones in the disease condition is still not clear. However, as per literature it is known that taurine is formed upon the breakdown of homocysteine known as the trans-sulfuration pathway by the action of folate, vitamin B12 and cysteine dioxygenase [34] and also through a cumulative effort of growth hormone (GH), signal transducers and activators of transcription 5 (STAT5) and vitamin B12 [35]. Studies have also shown an antagonism between taurine and homocysteine reflected by the rise of taurine levels in the body through a decrease of homocysteine levels [36]. This inverse role of taurine to homocysteine also ties into the fact that taurine is a compound which has been shown to inhibit adipogenesis in white adipose tissue through the downregulation of genes related to adipogenesis such as PPARγ [37], reinstating a possible correlation of the two compounds with the actions of PPARγ upon binding to the LBD.

Given these considerations, the purpose of this study was to examine the likely molecular mechanism/s by which taurine and homocysteine interact with PPARγ in relation to osteoporosis. Our specific goal was to find out how taurine and homocysteine affect PPARγ activity, as well as its effects on osteoclastogenesis and osteogenesis. Furthermore, as previously mentioned, vitamin B12 has been demonstrated to be essential in raising taurine and lowering homocysteine levels in prior studies. Therefore, it was included in this study to learn more about its function in preventing homocysteine-induced osteoporosis both independently and in combination with taurine. Using both *in-silico* and *in-vitro* analyses, this study aims to enhance understanding of the mitigation of osteoporosis in hyperhomocysteinemic conditions and potentially open up new therapeutic opportunities.

## Methodology

The investigation was carried out using an HP Pavilion laptop with a 64-bit Windows 11 operating system and an 11th Gen Intel(R) Core (TM) i5 processor with 4 Core(s) and 8 Logical Processor(s) with 4GB NVIDIA GeForce GTX 1650 and 4GB Intel(R) Iris(R) Xe GPU support. In addition, a portion of the molecular docking investigation was conducted using the Ubuntu 20.04.4 LTS subsystem of Windows 11.

### Study design

**Fig. 2:**
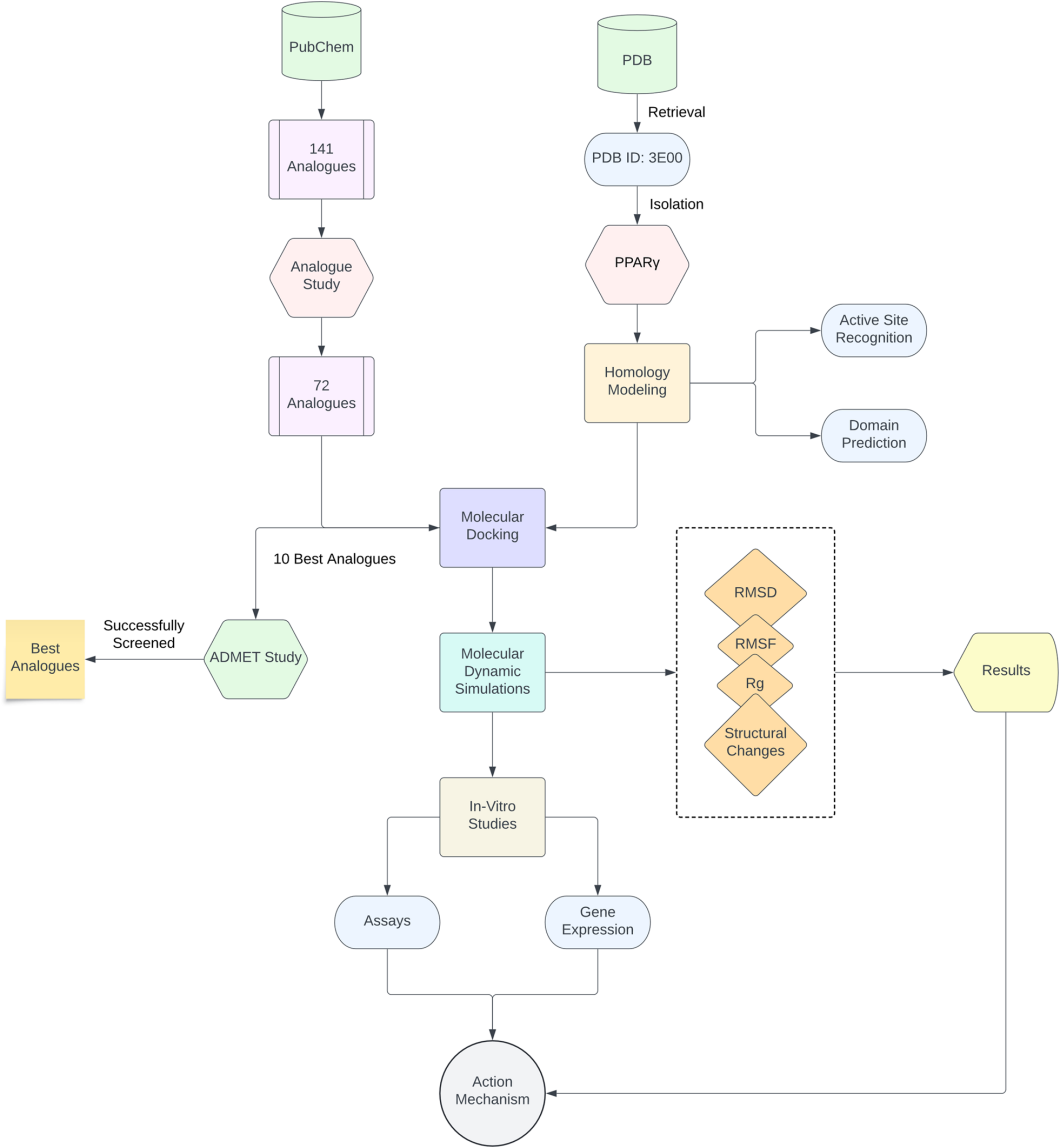
The figure illustrates the study design that was followed for drawing conclusions to this study performed via the integrative *in-silico* and *in-vitro* approach

The workflow for the study is depicted in Fig. 2. Initially, the human PPARγ protein sequence was obtained from the National Center for Biotechnology Information (NCBI) and blasted by using BLASTp to search for the best template structure available in the protein data bank (PDB) database (https://www.rcsb.org/) based on the identity and coverage scores to build the protein homology with. The homology modeling was then performed and optimized using the SWISS-MODEL (https://swissmodel.expasy.org/) server and used subsequently for molecular docking. The docking grid was then defined for docking analyses using the homology modelled structure of PPARγ following which it was docked with the ligands used for the study including two compounds of interest. The molecular dynamic simulation of the best docked poses was performed to identify the various probable binding configurations of the compounds and their potential for interacting with essential residues to instigate a probable agonistic or antagonistic change in the structure of the protein. This was followed by *in-vitro* analysis of the *in-silico* inferences using assays and gene expression studies with the compounds—taurine, homocysteine and vitamin B12. Ultimately, molecular docking was performed with a set of 72 taurine analogues obtained from PubChem (https://pubchem.ncbi.nlm.nig.gov/) using a Tanimoto threshold of 90% after excluding 62 unconjugated analogues against the PPARγ receptor, followed by the selection of the top ten results based on the binding energy obtained from the analogue study. This selection was then evaluated by analyzing the compounds' thorough absorption, distribution, metabolism, excretion and toxicology (ADMET) reports.

### Homology modeling

Initially, the sequence of the human PPARγ was downloaded from the NCBI (Accession: P37231.3). The available template for it was searched on PDB using the BLASTp program of NCBI with the default parameters which were then selected based on identity and coverage scores. The FASTA sequence of this template model was then retrieved from the PDB (PDB ID: 3E00) and homology was modeled using SWISS-MODEL [38] to eliminate any false data arising from missing atoms in the original PDB structure. The homology model was retrieved from SWISS-MODEL and set aside for performing molecular docking.

### Domain prediction

To predict domains present in the modeled PPARγ protein, the FASTA sequence from PDB was submitted to InterPro [39] server (https://www.ebi.ac.uk/interpro/) and SMART [40, 41] server (https://smart.embl-heidelberg.de/) which predicts the myriad signatures of a protein such as the family to which it belongs, the domains having the best conservation and the sites of interest to consider in the protein while studying its functionality.

### Molecular docking

#### Preparation of protein

The homology-modeled PPARγ structure was then set up for docking using MGL Tools-1.5.7, Autodock 4.2.6, and Biovia Discovery Studio 2021 Visualizer. To prepare the protein for use as a docking target, the attached ligand and metals were extracted from the complex using Discovery Studio 2021 Visualizer. Polar hydrogen bonds and Kollman charges were then added to the complex using Autodock 4.2.6 workspace, and the protein was saved in the PDBQT format.

#### Active site determination

The active site of the modeled protein was determined by using the Proteins Plus [42, 43] server (https://proteins.plus/) with the help of its DoGSiteScorer [44, 45] module and the most druggable pocket was noted for performing the docking of our compounds of interest. This active site was then confirmed by using the CASTp 3.0 [46] server (http://sts.bioe.uic.edu/castp/).

#### Ligand retrieval and preparation

In this study, the two main ligands of interest—taurine and homocysteine were retrieved from PubChem. The other three agonists, two antagonists, and one inverse agonist were retrieved from PDB. Table 1 shows the PDB IDs from which they were retrieved for more information. The ligands were then prepared for docking by energy minimization for 1000 steps by applying Merck Molecular Force Field (MMFF94) before saving them in the PDBQT format using Open Babel. The number of rotatable bonds in the ligand was not changed or modified. This is the precise process that was employed to prepare the taurine analogues for docking with the target protein.

**Table 1:** List of the ligands used in this study

Name of Ligand	Role	PDB ID	2-D Structure
EDK	Agonist	6D8X	
Hydroxy Pioglitazone	Agonist	6DHA	
Rosiglitazone	Agonist	5YCP	
14R	Antagonist	4HEE	
SR11023	Antagonist	6C5T	
SR10171	Inverse Agonist	6C5Q	
Taurine		PubChem Structure	
Homocysteine		PubChem Structure	

The ligands have been specified along with the PDB IDs from which they were isolated except for taurine and homocysteine which were retrieved from PubChem

#### Ligand docking

Once the ligand and the protein receptor had been prepared, the molecular docking process was performed with the help of the Autodock Vina (ADV) program [47]. The grid box was prepared in the active pocket which had been identified previously and the exhaustiveness of the docking to be performed was set to 32. The dimensions of the grid for the X, Y, and Z dimensions were set to 40 Å each while the X, Y, and Z center coordinates were set to -9.779053 Å, 18.508211 Å, and 14.547474 Å respectively for all the docking runs. For the docking of each ligand with the receptor, 10 binding modes were obtained out of which the best was selected for further analysis based on the binding affinity and the RMSD values.

### Molecular dynamic simulation

The poses found to have the highest binding affinity from the docking simulations i.e., the protein–ligand complexes were chosen to undergo molecular dynamic simulations (MD simulations) using the Nanoscale Molecular Dynamics (NAMD) software program [48]. The purpose of running the simulations was to determine the stability with which the compounds of interest would most certainly bind with the receptor and how favorable these interactions would be towards the understanding of the binding patterns while also identifying the binding residues in the receptor that associate themselves with the interactions in terms of its occupancy. The docked coordinate-oriented parameterization of the compounds under study was performed using the SwissParam web server [49] which applies the CHARMM all atoms force field. The CHARMM force field parameters were also used to describe the receptor PPARγ before preparing the input files which would be used for energy minimization, dynamics, and analysis using the Visual Molecular Dynamics (VMD) [50] software program. The solvation for all the systems was performed in a TIP3P water box with a padding of 5 Å edges from the protein–ligand complexes and the system was neutralized with NaCl. The system was minimized using the steepest descent algorithm for 10,000 steps after which the simulation was run for a period of 10 ns. The trajectory coordinates and the energy were collected every 0.01 ns and 0.002 ns respectively with a timestep of 2 fs.

### *In-vitro* studies

#### Chemicals and reagents

The human osteoblast-like Saos-2 cells and the mouse macrophage cell line RAW 264.7 were obtained from the National Centre for Cell Sciences (NCCS, Pune, India). DL-Homocysteine was acquired from the Tokyo Chemical Industry (TCI, Japan). Taurine, 2′,7′-dichlorofluorescin diacetate (DCF-DA) probe, Griess’ reagent, Lipopolysaccharides (LPS) from E. coli, and qPCR primers were purchased from Sigma-Aldrich (India). The Oligo (dT) Primer was acquired from GeNei (India). Vitamin B12, Thiazolyl Blue Tetrazolium Bromide (MTT), and BioLit FluroGreen qPCR Master Mix from the Sisco Research Laboratories (SRL, Mumbai, India). RNAiso Plus and PrimeScript™ Reverse Transcriptase were obtained from Takara, Japan. sRANKL was obtained from PeproTech (USA). Cell culture reagents including Dulbecco’s Modified Eagle’s Medium (DMEM), fetal bovine serum (FBS), antibiotic–antimycotic solution, Alizarin Red stain, ascorbic acid and 2,2-diphenyl-1-picrylhydrazyl (DPPH) was purchased from Hi-Media laboratories, Mumbai, India.

#### Cell culture

The RAW 264.7 and the human osteoblast-like Saos-2 cells were cultured in DMEM supplemented with 10% FBS and 1% antibiotic–antimycotic solution in a humidified incubator at 37°C and 5% CO_2_. Cells were extracted and studied after they reached 80–90% confluence in a sterile culture flask.

#### Cell viability assay

The cell viability assay was performed with undifferentiated RAW 264.7 cells. The cells were seeded at a density of 1 × 10^4^ cells per well supplemented with complete media in a 96-well plate and incubated overnight in a humidified incubator at 37°C and 5% CO_2_. The cells were treated the following day with taurine, homocysteine, and vitamin B12 at varying concentrations for 24 and 48 h. After the incubation period, the spent media were aspirated, and MTT (0.5 mg/ml final concentration) was added to each well. The plate was then incubated in the dark for 3 h at 37 °C. Post the 3 h of incubation, the MTT from the wells was removed, 100 µL DMSO was introduced and the absorbance was measured at 570 nm by a microplate reader (BioTek, USA) to quantify the viable cells as described before [51]. The viability of the cells upon treatment with the different ranges of hydrogen peroxide (H_2_O_2_) was also assessed for subsequent cytoprotective assays.

#### Cytoprotective assay

The cytoprotective assay was also performed with undifferentiated RAW 264.7 cells. The cells were seeded at a density of 1 × 10^4^ cells per well supplemented with complete media in a 96-well plate and incubated overnight in a humidified incubator at 37°C and 5% CO_2_. Based on the results of the cell viability assay three concentrations were selected which were used for treating the cells the following day and then incubated for the next 5 h. Post incubation, the treatment media was aspirated and then substituted with the chosen H_2_O_2_ concentration obtained from the cell viability assay for the next 19 h. Following the incubation time, MTT (final concentration of 0.5 mg/ml) was introduced to each well after the spent media were aspirated. After that, the plate was incubated for three hours at 37 °C in the dark. Following a three-hour incubation period, the MTT was extracted from each well, 100 µL of DMSO was added, and a microplate reader (BioTek, USA) was used to detect the absorbance at 570 nm to quantify the viable cells.

#### Cytotoxicity protection assay

The cytotoxicity protection assay was conducted as well on undifferentiated RAW 264.7 cells. The cells were seeded at a density of 1 × 10^4^ cells per well on a 96-well plate supplemented with complete medium and incubated overnight in a humidified incubator at 37°C with 5% CO_2_. The cells were treated the following day with select concentrations of taurine and vitamin B12 and subsequently incubated for 5 h. After the incubation time, the treatment media was replaced with a cytotoxic dose of homocysteine which was then incubated for the next 19 h. The spent media were aspirated and MTT (final concentration of 0.5 mg/ml) was added to each well after the incubation period. Subsequently, the plate was kept in the dark at 37°C for 3 h. After incubating for 3 h, the MTT in each well was removed, 100 µL of DMSO was added, and the absorbance at 570 nm was measured using a microplate reader (BioTek, USA) to quantify the viable cells.

#### Reactive oxygen species (ROS) assay

The reactive oxygen species (ROS) assay was used to determine the intracellular ROS scavenging capability of the compounds of interest in undifferentiated RAW 264.7 cells. The cells were seeded at a density of 5 × 10^5^ cells per well on a 12-well plate with complete media and incubated overnight in a humidified incubator at 37 °C and 5% CO_2_. The cells were treated with the chosen compound dosages of taurine, homocysteine, and vitamin B12 for 24 h. The cells were then washed in phosphate-buffered saline (PBS) before being extracted from each well using trypsin.

To investigate the effect of treatment concentrations on reducing the ROS levels in activated macrophages, cells were treated with 1 µg/ml LPS for 2 h after the initial overnight incubation. Following that, the compounds and their combinations were added to the LPS-containing medium and incubated for an additional 24 h. The cells were then rinsed with PBS before being extracted from each well using trypsin.

The cells that were extracted were treated with 20 µM DCF-DA dye for 30 min in the dark. ROS levels were quantified using a spectrofluorometer (FP-8200 JASCO, UK) and validated using a flow cytometer (CytoFLEX, Beckman Coulter, USA) at the emission wavelength of 530 nm and the excitation wavelength of 485 nm.

#### Cell differentiation

To obtain mature osteoblast-like cells, the Saos-2 cells were cultured in osteogenic media consisting of DMEM supplemented with 10% FBS, 1% antibiotic–antimycotic solution, 50 µg/ml of L-ascorbic acid, and 10 mM of β-glycerophosphate. The media was changed every 2–3 days. The cells were treated with drugs for 7 and 14 days during this time. The cells were incubated in a humidified incubator at 37 °C and 5% CO_2_.

The RAW 264.7 cells were differentiated into osteoclasts by incubating them in an osteoclast induction medium containing 50 ng/ml of RANKL in DMEM supplemented with 10% FBS and 1% antibiotic–antimycotic solution. The cells were then cultured for 5 days. The morphology of the osteoclasts were validated using May-Grünwald Giemsa staining [52, 53]. The osteoclast induction medium was additionally supplemented with homocysteine to better understand its involvement in osteoclastogenesis, and the investigation was carried out as previously described, with a change of wasted media every two days. On the last day, the compounds of interest were given to the osteoclasts, both individually and in combination, and incubated for an additional 24 h. The cells were incubated in a humidified incubator at 37 °C and 5% CO_2_.

#### Nitric oxide (NO) assay

The undifferentiated Saos-2 cells were plated at a density of 1 × 10^4^ cells/well in 12-well plates and incubated overnight at 37 °C in a 5% CO_2_ incubator. The media were then removed and the cells were treated with the selected dosages of the compounds in osteogenic media for the next 7 days. After 7 days of incubation, the nitrite level in the cell culture media was determined using the Griess’ reagent. Briefly, 100 µL of cell culture medium was added to 100 µL of Griess' reagent and incubated for 15 min at room temperature. A microplate reader (BioTek, USA) was then used to measure the absorbance at 540 nm.

#### Alizarin red stain (ALS) assay

To determine the amount of mineralization in the differentiated Saos-2 cells, alizarin red staining was performed. The undifferentiated Saos-2 cells were seeded at a density of 1 × 10^4^ cells per well in a 46-well plate and incubated overnight in a humidified incubator at 37 °C and 5% CO_2_. The following day, the spent media for cells were substituted with selected dosages of compounds in osteogenic media and incubated for 7 and 14 days. Post 7 and 14 days, the differentiated cells treated with the treatment doses were washed with PBS and fixed in 200 µL of 10% (v/v) formaldehyde for 15 min. After washing the cells twice with distilled water, 200 µL of 40 mM ALS was added and incubated for 20 min at room temperature while gently shaking. Excess stains were removed from the plates by washing them with distilled water for better visualization under an inverted microscope (INVI, Magnus, India). Briefly, the ALS stain was assessed for mineralization by calcium deposition. For this, 100 µL of 10% acetic acid was added to the wells and incubated at room temperature for 30 min. The cells were scraped and transferred into a tube, and vortexed vigorously for 30 s. Thereafter, the tubes were incubated at 85 °C for 10 min followed by a quick transfer to ice for 5 min. The resulting slurry was centrifuged at 12,000 rpm for 15 min before being transferred to a 96-well plate and measured for absorbance at 405 nm with a microplate reader (BioTek, USA).

#### Gene expression analysis

The RAW 264.7 cells were stimulated with LPS and treated with the selected treatment dosages as described previously. The differentiated Saos-2 and RAW 264.7 induced osteoclast cells were also treated as aforementioned. After the completion of the treatment period, the mRNA was extracted using RNAiso Plus. RNA was quantified using Nanodrop (NanoDrop™ One, Thermo Scientific). RNA was transcribed to cDNA using PrimeScript™ Reverse Transcript, then amplified with BioLit FluroGreen qPCR Master Mix and suitable forward and reverse primers (Table 2). The 18S gene was used as an endogenous control. The 2^−ΔΔCq^ technique was utilized to determine the results. The final results were expressed as the fold change relative to control.

**Table 2:** Sequences for primers used for qPCR experiments

Gene	Forward sequence (5'–3')	Reverse sequence (5'–3')
18S	CTCAACACGGGAAACCTCAC	CGCTCCACCAACTAAGAACG
PPARγ	TGGCCCACCAACTTCGGAAT	CCCACAGACTCGGCACTCAA
SOD1	ACCTGGGCAATGTGACTGCT	CAAGCGGCTCCCAGCATTTC
SOST	GGTGGCAAGCCTTCAGGAAT	TGGGGAGGTCTGCCTCCATT
DKK1	CCCTACCCTTGCGCTGAAGA	GGGAGCCTTTCCGTTTGTGC

The primers used in the study have been listed spanning in the direction of 5’ to 3’

#### Statistical analysis

The statistical analysis was performed using GraphPad Prism 9.0, and all experiments were repeated at least three times. Data is provided as mean ± standard error mean (SEM). A two-way analysis of variance with Bonferroni's correction was conducted to examine group differences, with *p* < 0.05 indicating significance.

### ADMET profiling

The SwissADME [54] web server (http://www.swissadme.ch/) was used to profile the top 10 compounds based on their highest binding energies with the target protein. Out of all factors determined by the SwissADME web server to provide an accurate and reliable profile of the compounds under investigation, the logarithm of the n-octanol/water distribution coefficients (LogP), water solubility (LogS), topological polar surface area (TPSA), gastrointestinal absorption (GIA), blood–brain barrier (BBB) permeation, skin permeation (SP), cytochrome P450 family inhibition (CYP-I), Lipinski's rule of five (LoF), PAINS alerts (PAINS) and synthetic accessibility (SA) were regarded as the major assessors that enable the removal of improbable compounds. The toxicity of the compounds was screened after ADME with the help of the ProTox-II [55] web server (http://tox.charite.de/protox_II/).

### Visualization of interactions

To visualize and analyze the docking results in the form of 2D or 3D interactions between the ligand and the protein post-docking, Discovery Studio 2021 Visualizer from Biovia, and UCSF ChimeraX have been used. The visualization and analysis of the results from the MD simulations were performed using VMD 1.9.3 and UCSF ChimeraX.

## Results

### Modeled homology analysis

Many structures of the PPARγ ligand binding domain (LBD) are available in the protein data bank (PDB) but most of those structures are not intact or have missing residues in them. Hence the intact PPARγ LBD was modeled for homology in the SWISS-MODEL server using the template PPARγ (PDB ID 3E00) to derive a 3D homology model for the LBD with a global model quality estimate (GMQE) of 0.79, QMEANDisCo global score of 0.74 ± 0.05 and a qualitative model energy analysis (QMEAN) Z-score of -2.47. Both the coverage-dependent scores obtained from GMQE and the non-explicit coverage-dependent scores from QMEANDisCo showed that the overall model quality is good. This was further confirmed by the scores obtained in QMEAN which is well within the typical standard deviation value for a reliable model. The model showcased a value closer to 1 for most of the residues which indicates that the overall local quality of the predicted model is good although a few of the residues did have values less than 0.6 which signifies low-quality modeling of those residues. The comparison plot showed that the model lies in the outliers of well-defined experimental structures but within the range of other protein structures in PDB, confirming its reliability as a model. The ‘MolProbity’ score was found to be 2.17 for the model while the Ramachandran plot obtained from the SWISS-MODEL implied that almost 89% of its residues were in the most favored regions and 1.90% of its residues resided in its outliers confirming it as a reliable model even though it might not be close to the crystallographic structure. The complete structure validation statistics along with the rest of the data obtained upon performing the modeling can be observed in Fig. S1.

### Domain recognition

InterPro is a domain identification and functional analysis tool that predicted the presence of two primary domains in the homology modeled PPARγ—the zinc finger, DNA binding domain (residues 50–143) and the nuclear hormone receptor, ligand binding domain (residues 152–417). It also predicted a few active site residues of the protein found through the functional analysis which has been depicted in Figure S2. Through this identification of domains, the ligand binding domain (LBD) was identified as the target for this study.

### Molecular docking analysis

**Fig. 3:**
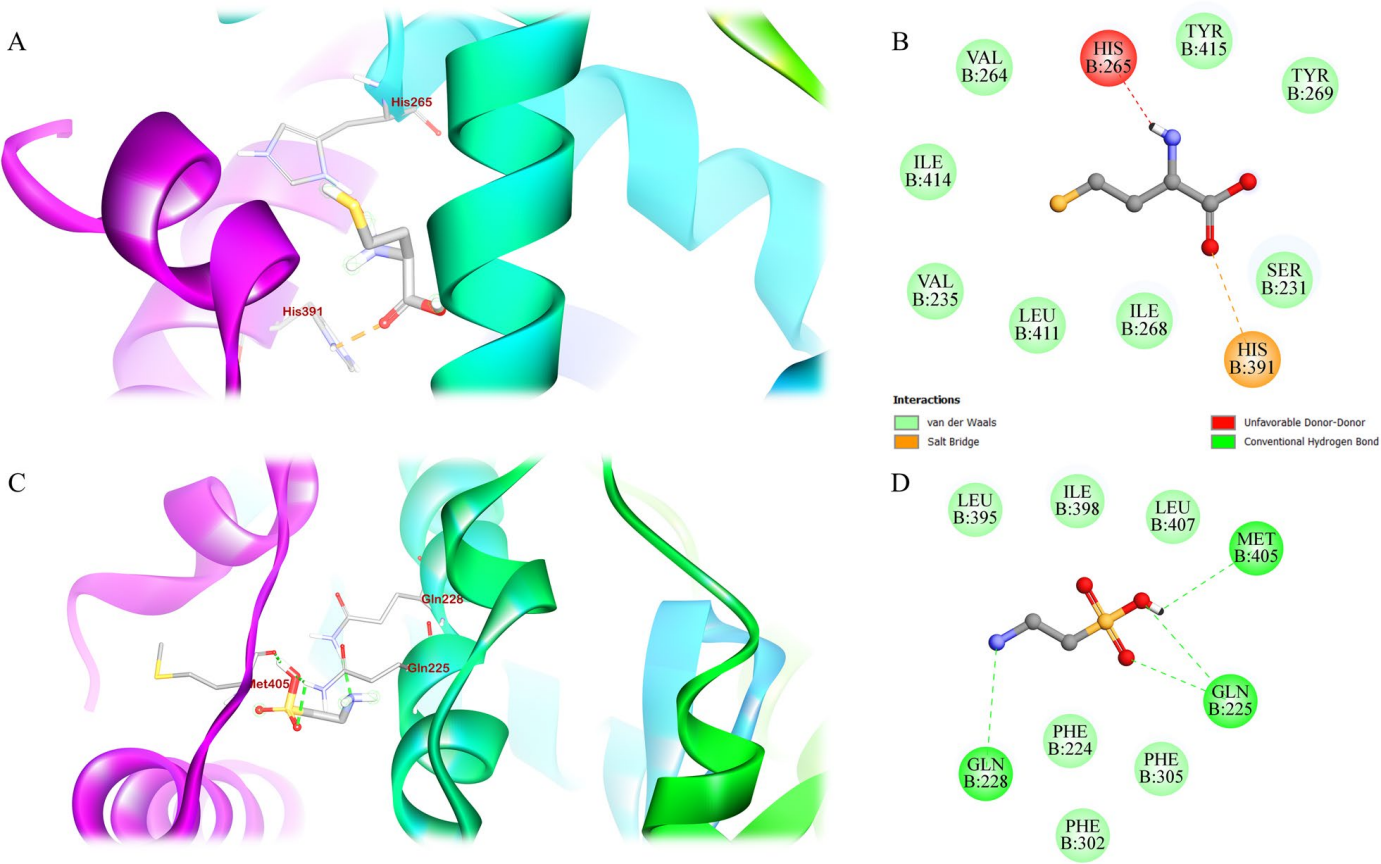
Molecular interactions of homocysteine and taurine against PPARγ LBD. Figures **A**, **B** depict the interaction with homocysteine, where **A** is the residue interactions of PPARγ with homocysteine, **B** is the 2-D representations of those interactions; **C**, **D** depict the interaction with taurine, where **C** is the residue interactions of PPARγ with taurine, and **D** are the 2-D representations of those interactions

Molecular docking of the ligands was performed in silico to ascertain the binding affinities and to understand the mechanism of interaction between the ligands and the modeled PPARγ receptor. The ligand GW9 which was supplied by SWISS-MODEL to mark the binding site of the modeled PPARγ was removed for docking purposes and the position was noted and verified for being the active site by using the CASTp server and Protein Plus server (Figure S3). The binding potential of the pocket was validated by docking that site with three agonists, two antagonists, and one inverse agonist (Figure S4). Upon performing the docking procedure, the binding poses of the two ligands of interest and their interactions with the residues were noted and can be seen in Fig. 3.

The binding affinities of each of the validation ligands are shown in Table S1 while the two ligands of interest obtained upon completion of the docking process are tabulated in Table 3 along with the various interactions observed with the binding residues. In contrast to homocysteine, which exhibited a salt bridge but no hydrogen bonding, three hydrogen bonds were seen with taurine. After the docking procedure was finished, the binding affinities of taurine and homocysteine were found to be −4.1 kcal/mol and −4 kcal/mol, respectively.

**Table 3:** The table depicts the binding affinity and residue interactions of the compounds of interest with the target

Name	Binding affinity (kcal/mol)	Hydrogen bond interactions	Hydrophobic interactions	Salt bridges interactions	Bound loops
H	−4	**	231S, 235 V, 264 V, 265H, 268I, 269Y, 411L, 414I, 415Y	391H	H3, H4-H5, H11 and H12
T	−4.1	225Q, 228Q, 405 M	224F, 302F, 305F, 395L, 398I, 407L	**	H3, H11 and H12

The interacting residues have also been listed in the tables along with the PPARγ loops involved. The absence of particular interactions is represented by ‘**’

### Study of molecular dynamic simulations

The molecular dynamic simulation study revealed the molecular stability of the selected docking complexes and their dynamic behavior in terms of the interactions with the PPARγ LBD residues over a time span of 100 ns. It was observed that upon individual analysis of the binding with homocysteine and taurine, homocysteine was stable in its docked groove but taurine started to shift from its initial docking groove after a few frames until it traversed quite some distance to a different spot. This was quite a fascinating lead which was also taken into account while designing further evaluations of taurine in its role in PPARγ. The root mean square deviation (RMSD), root mean square fluctuations (RMSF), radius of gyration (Rg), and the number of hydrogen bond interactions after the completion of the 100 ns simulation gave the needed deeper insight into the roles of the two compounds and their probable overall action for mediation of its agonistic or antagonistic properties.

#### Root mean square deviation (RMSD) analysis

**Fig. 4:**
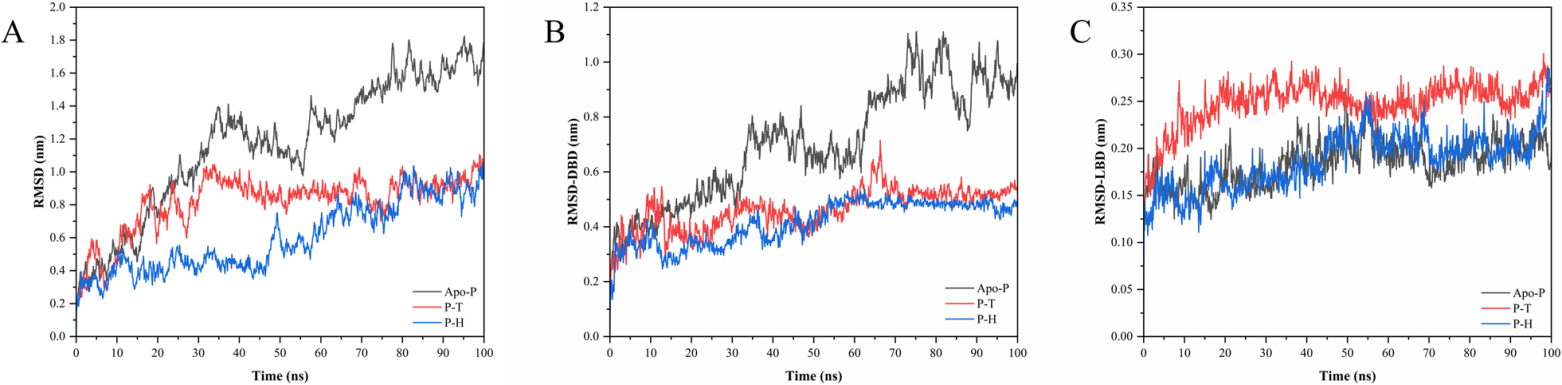
The figure shows the results of the MD simulation trajectory. The plots in figure **A**- **C** represent the **A** RMSD of the apo-PPARγ (Apo-P), taurine-bound PPARγ (P–T), and homocysteine-bound PPARγ (P–H). The RMSD of **B** the DNA binding domain (DBD) and **C** the ligand binding domain (LBD) of PPARγ was also analyzed separately for the apo-PPARγ and compounds bound to PPARγ

To investigate the dynamics and stability of the best docked conformer from the docked complexes, an RMSD analysis was performed. RMSD is considered an integral and important parameter to analyze the trajectories obtained from MD simulations. The RMSD values were estimated for the backbone atoms of the docked complexes and the measurements of these provided the necessary insights into the conformational stability. The comparisons of the RMSD values for taurine-bound and homocysteine-bound PPARγ are shown in Fig. 4[A–C]. It was observed that the average RMSD value of the apo- PPARγ was 1.17 nm while the RMSD value of PPARγ bound with homocysteine was found to be 0.60 nm and that of PPARγ LBD bound with taurine was found to be 0.82 nm. The RMSD values here are inclusive of the highly flexible DBD and highly stable LBD regions, and hence the overall structures as seen in are seen to be more stabilized by the binding of the compounds. As can be seen from Fig. 4[B], the addition of the compounds stabilizes the DBD with homocysteine-bound PPARγ showing more stability in comparison to taurine-bound PPARγ. In contrast to DBD, in the LBD region of the target protein, it can be observed that taurine affects the structural integrity of PPARγ more in comparison to homocysteine (Fig. 4[C]). A very big structural change can be seen in the LBD of PPARγ bound with taurine almost throughout the 100 ns simulation. The PPARγ LBD bound with homocysteine shows no significant structural changes and the RMSD values can be observed to be well below that of the apo-PPARγ RMSD. Based on these results from the RMSD analysis, it can be deduced that the stability of homocysteine-bound PPARγ is much more than that of taurine-bound PPARγ however taurine disrupts LBD structural integrity more than homocysteine.

#### Root mean square fluctuation (RMSF) analysis

**Fig. 5:**
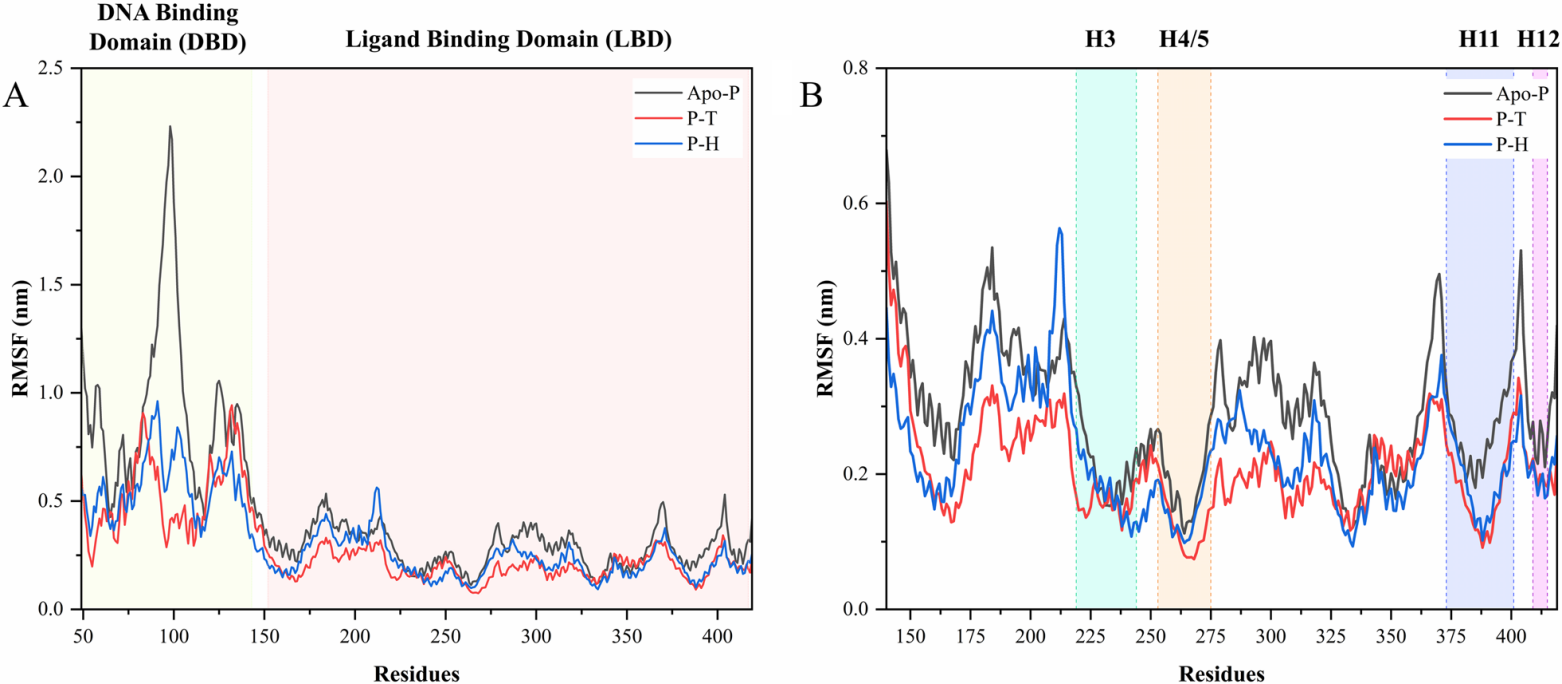
The figure shows the results of the MD simulation trajectory. The plots in figure **A** RMSF graphs of apo-PPARγ (Apo-P), taurine bound PPARγ (P–T) and homocysteine bound PPARγ (P–H), **B** RMSF graph of the PPARγ LBD and apo- and holo-forms with the four important helices involves in the activation and action of PPARγ. The cyan color in the graph represents Helix 3 (H3), the orange represents Helix 4/5 (H4/5), the blue represents Helix 11 (H11) and the purple color represents Helix 12 (H12) of PPARγ

The flexibility of the apo- and holo-PPARγ's backbone structures was estimated using RMSF analysis. High RMSF values are known to indicate increased flexibility of the amino acid residues in the protein, which account for the majority of the protein's molecular movements. Figure 5[A] compares the RMSF values for taurine and homocysteine-bound PPARγ. The residues 50–143 form the unbound DNA binding domain (DBD), hence they fluctuate considerably over the 100 ns simulation in the apo-protein, as expected. However, the holo-proteins seem to have more stabilized fluctuation in this region as can be observed from the plot as well. All four key helices involved in PPARγ activation show a significant loss in flexibility, with significant structural changes likely following the binding of taurine and homocysteine to the LBD of PPARγ. Figure 5[B] shows the PPARγ LBD–specific RMSF plot.

#### Radius of gyration (Rg) analysis

**Fig. 6:**
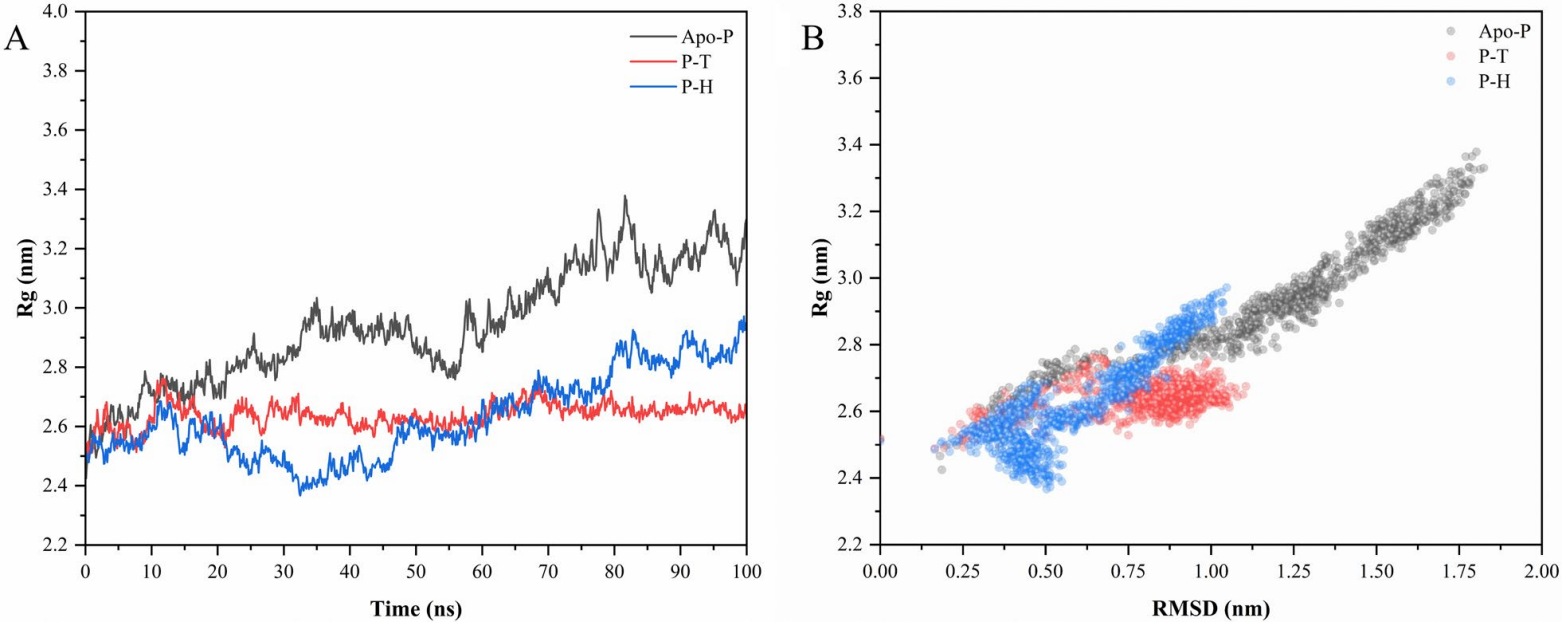
The figure shows the results of the MD simulation trajectory. The plots in figure **A** Rg graphs of apo-PPARγ (Apo-P), taurine bound PPARγ (P–T) and homocysteine bound PPARγ (P–H), **B** RMSD vs Rg plot for the entire 100 ns of simulation

Rg analysis is crucial for determining the impact of ligands on protein folding and compactness since it correlates with changes in the protein's tertiary structure or natural conformation. The compactness of the apo- PPARγ and PPARγ bound to the compounds of interest was determined by assessing the Rg plot, as shown in Fig. 6[A]. The plot shows that the apo- PPARγ is less compact than the halo-form. The same plot shows that the PPARγ bound with homocysteine mimics the variations in the apo-PPARγ between 60 and 100 ns, indicating that homocysteine was able to further stabilize the apo-PPARγ backbone upon binding. In the instance of taurine-bound PPARγ, the variations were minimal and nearly reached a plateau phase, indicating that taurine provided stability to the PPARγ backbone. This is further reinforced in Fig. 6[B] by the plotting of Rg values against the RMSD values for the 100 ns simulation run. The results of this study show that when apo-PPARγ binds to taurine and homocysteine, it stabilizes its structure by inducing significant tertiary compaction. The modifications in the tertiary structure of the halo-PPARγ appear to be consistent with the results from the RMSD and RMSF studies.

#### The hydrogen bond pattern analysis

**Fig. 7:**
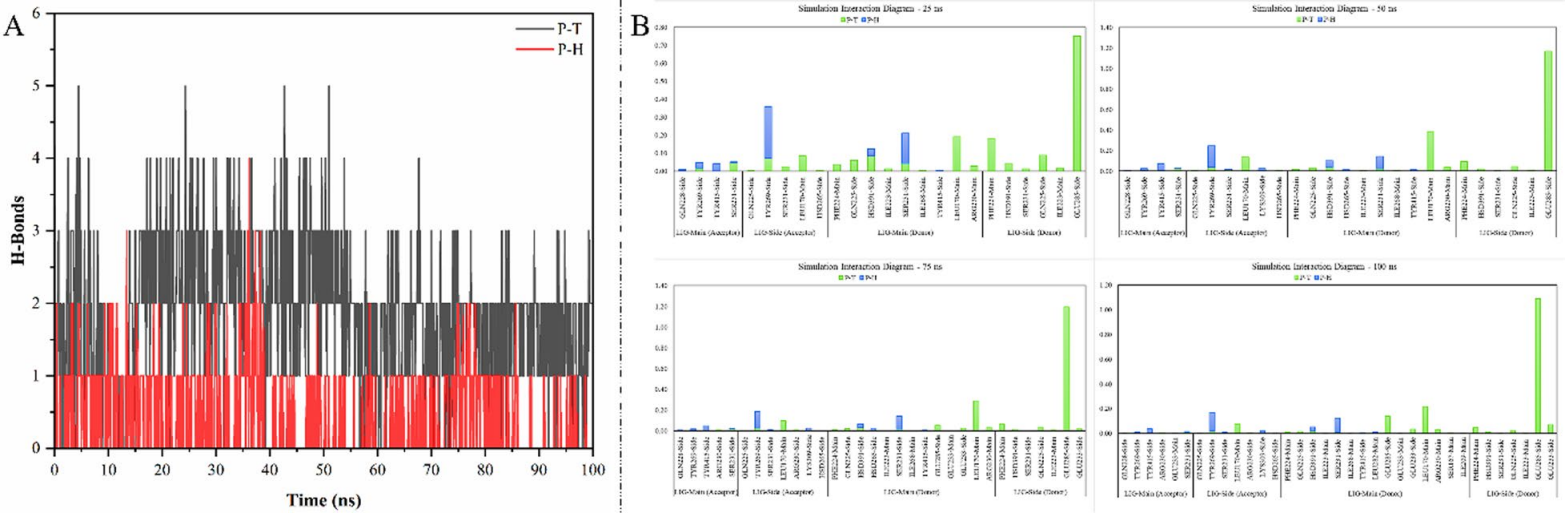
The figure shows the results of the MD simulation trajectory. **A** the total number of intermolecular H-Bonds between—PPARγ LBD and taurine (P–T) and PPARγ LBD and homocysteine (P–H), **B** Simulation interaction Diagram (SID) as per the data of occupancy in terms of hydrogen bonds obtained after the 100 ns simulation and broken up to emulate a dynamic chart for the hydrogen bonds and its occupancy after every 25 ns

The arrangement of intermolecular hydrogen bonds in every protein–ligand system is critical because it aids in identifying the residues of interest and stabilizing a ligand during its interactions with the target protein. As a consequence, a hydrogen bond pattern analysis over a 100 ns period was undertaken to provide more context to the preceding results provided in the relevant sections. This pattern analysis has been shown in Fig. 7[A]. Mostly throughout the 100 ns simulation, it can be observed that taurine maintains a higher number of intermolecular hydrogen bonds with PPARγ LBD in comparison to homocysteine. This supports the considerable RMSD shifts in the LBD backbone of taurine-bound PPARγ over the simulation period. The hydrogen bond patterns of taurine-bound PPARγ having an overall higher number of intermolecular hydrogen bonds might also be the reason for it having lesser RMSF. A simulation interaction diagram was prepared based on the hydrogen bonds established and their occupancy with the interaction residues, as shown in Fig. 7[B]. These were measured at 25 ns intervals to further understand the dynamic nature of taurine and homocysteine interactions when bound independently to PPARγ LBD. After 100 ns, four residues were found to be shared by homocysteine and taurine when bound individually to the LBD of PPARγ. The residues were 228Q, 231S, 269Y, and 391H. The most significant interaction between the two compounds under investigation was by far taurine with 285E of PPARγ LBD, which had an occupancy of 122.88% after the 100 ns simulation.

#### Significant changes in the structure over the simulation

**Fig. 8:**
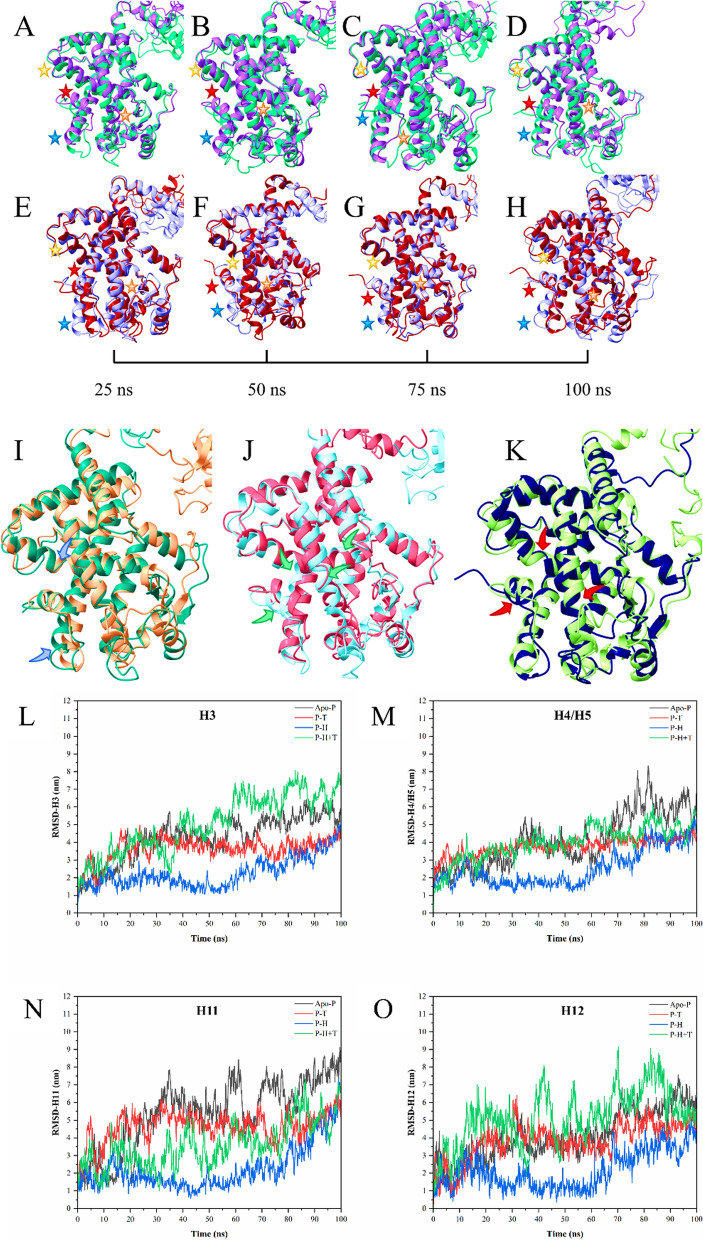
A representation of the structural changes occurring throughout 100 ns simulation. **A**–**D** Structural changes between taurine bound PPARγ (violet) and homocysteine bound PPARγ (cyan) over 25–100 ns time frame; **E–H** Structural changes between homocysteine bound PPARγ (lilac) and PPARγ bound with both taurine and homocysteine (maroon) over 25–100 ns time frame; **I** Changes in the structure of homocysteine bound PPARγ (P–H) during the 0 ns (yellow) time frame and 100 ns (green) time frames; **J** Changes in the structure of taurine bound PPARγ (P–T) during the 0 ns (blue) time frame and 100 ns (pink) time frames; **K** Changes in the structure of taurine and homocysteine bound PPARγ (P-H-T) during the 0 ns (green) time frame and 100 ns (blue) time frames; **L–O** RMSD graphs for **L** helix 3 (H3), **M** helix 4-helix 5 (H4/H5), **N** helix 11 (H11) and **O** helix 12 (H12) plotted with apo-PPARγ (Apo) helices. Red stars in the representation A-H signify structural changes in H12, blue stars show the changes occurring in H11, green stars notate the changes in H4 and orange stars mark the changes in H3 of PPARγ. The blue arrows indicate the changes in the helices between 0 and 100 ns structures of homocysteine bound PPARγ, the green arrows show the changes in the helices between the 0 ns and 100 ns structures of taurine bound PPARγ while the red arrow marks the prominent changes in between 0 and 100 ns structures of homocysteine bound PPARγ

Due to the dynamic nature of taurine, it was important to simulate homocysteine and taurine together to determine whether taurine has two separate effects when working alone and when working against or with some other compound. Since the binding affinity of taurine is much more than that of homocysteine and the initial binding pocket of both these compounds are different, it was only valid to simulate them both trying to bind with their respective binding pockets of the PPARγ LBD and upon the completion of the 100 ns simulation run, draw inference from it. As shown in Fig. 8 [A–H], there are several structural changes when PPARγ is bound to taurine and homocysteine individually. The most obvious examples include taurine-bound PPARγ, where there is a large increase in RMSD of the H12 associated with PPARγ activation (Fig. 8 [L–O]). The increased RMSD of taurine-bound PPARγ might be attributed to the interaction between taurine and 224F, 228Q, 231S, 233E, 269Y, and 391H residues in the LBD's H3, H4/H5, and H11 during the 100 ns simulation. The interactions of H3, H4/H5, and H11 helices residues with taurine may alter H12's conformational equilibrium, which is required for PPARγ activation. Due to taurine clashing sterically with 223I, 224F, 225Q, and 228Q over a 100 ns period, destabilization of H3 of PPARγ LBD can be observed. The alterations in H12 might potentially be caused by taurine interacting with 285E, pinching H3 towards the β-sheets. Homocysteine bound PPARγ on the contrary doesn’t show a lot of significant changes although some structural changes in the H4/H5 and H11 can be observed. The RMSD is quite low compared to that of the RMSD of apo-PPARγ helices. Figure 8 [E–H] also represents the action of taurine when homocysteine is already present in its binding pocket in contrast to when homocysteine was bound individually to PPARγ. It can be observed that taurine when bound with homocysteine affects mostly the H3 and H12 helices as can be confirmed from the RMSD plots. It also to some extent affects the H4/H5 of the helices as is seen reflected in the structural changes. The changes noticed in the structures due to the binding of these ligands at 0 ns and 100 ns are also as shown in Fig. 8 [I-K].

### *In-vitro* study analysis

#### Selection of treatment concentrations

**Fig. 9:**
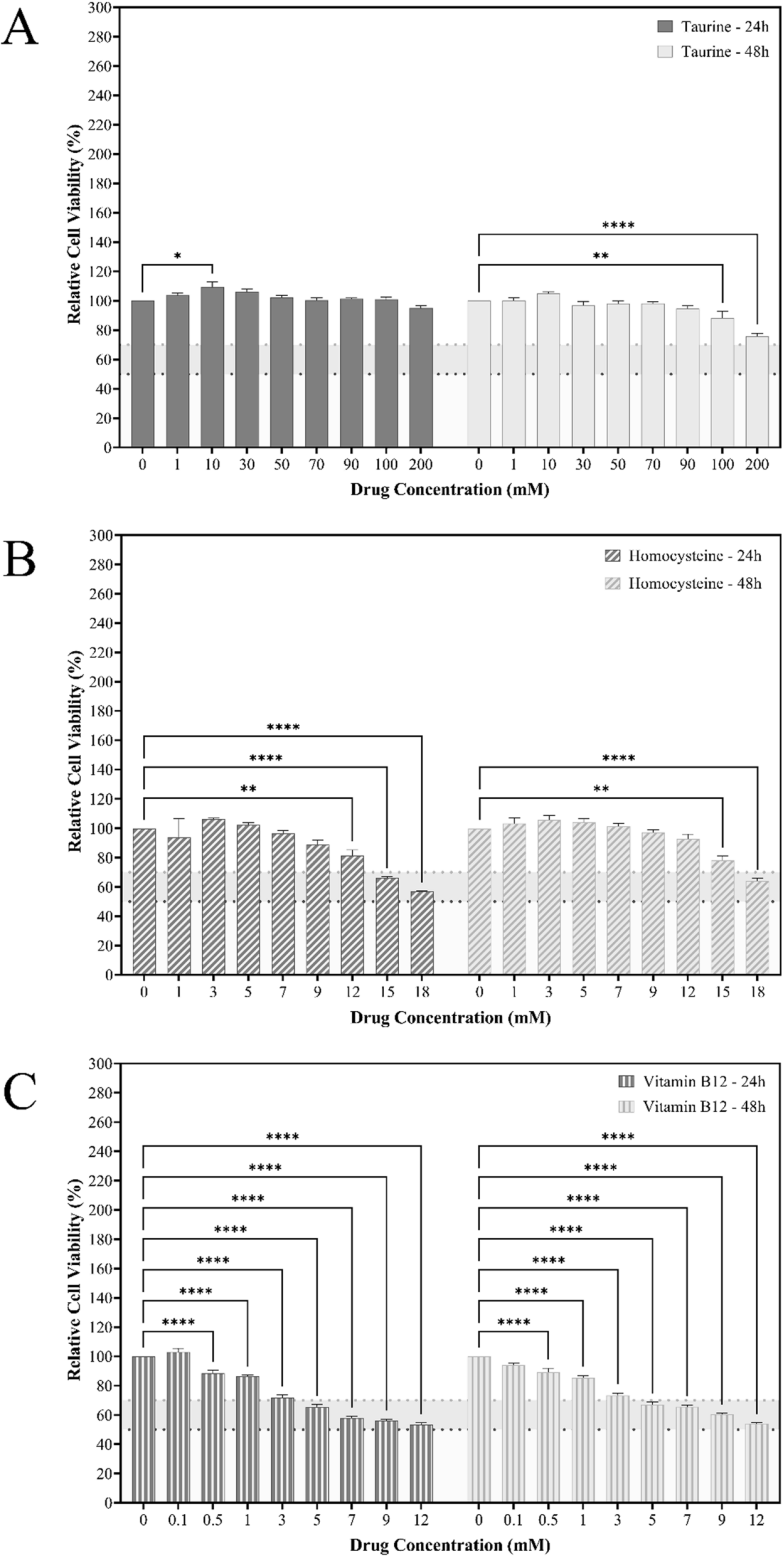
Representations of *in-vitro* assays performed on RAW 264.7 cells. **A–C** Demonstrates the effects of **A** taurine, **B** homocysteine, and **C** vitamin B12 on the cell viability. The results are expressed as mean ± SEM (n = 3)

The cell viability of RAW 264.7 macrophages treated with different concentrations of taurine (1–200 mM), homocysteine (1–18 mM), and vitamin B12 (0.1–12 mM) was assessed using an MTT test over 24 and 48 h, as shown in Fig. 9. With increasing taurine concentrations, cell viability began to diminish. This was more subtle in the first 24 h, but the changes in viability became more apparent during the next 48. Even then, the dosages kept the cell viability over 70%. However, in the case of homocysteine, the first 24 h indicated a dose-dependent decrease in cell viability, but after 48 h, the cell viability appeared to increase. In the instance of vitamin B12, we detect a dose-dependent reduction in cell viability in the first 24 h, which does not alter appreciably in the subsequent 24 h after vitamin B12 treatment of the cells. The experiment yielded taurine doses of 50, 100, and 200 mM, as well as homocysteine concentrations of 9, 12, and 15 mM as encompassing dosages for future studies. The test also enabled the selection of vitamin B12 doses of 0.5, 1, and 3 mM. The viability of RAW 264.7 cells treated with various concentrations of H_2_O_2_ (0.1–5 mM) was also evaluated in order to produce cytotoxic and stressful conditions inside the cells, as shown in Figure S5. Based on the results obtained from the analysis, 0.9 mM H_2_O_2_ was chosen for subsequent experiments as it showed only a 50% cell viability at the conclusion of the assay.

#### Investigation of the potential of compounds to ameliorate cytotoxicity induced by H_2_O_2_ and homocysteine

**Fig. 10:**
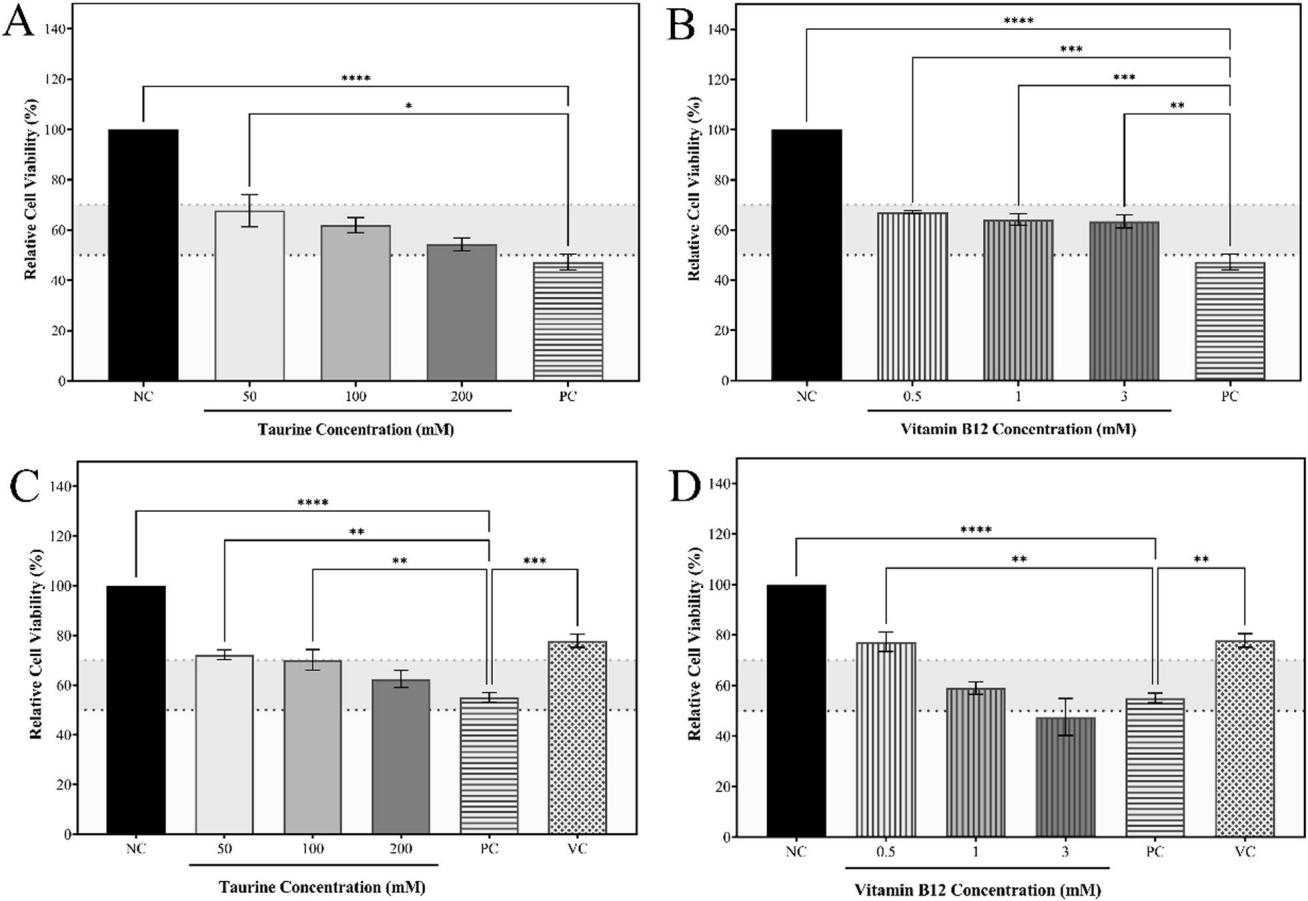
Representations of *in-vitro* assays performed on RAW 264.7 cells. The panels **A**, **B** shows the graphs obtained upon analysis of the cytoprotective effects of **A** taurine, and **B** vitamin B12 against H_2_O_2_-induced cytotoxicity. The panels **C**, **D** represents the graphs obtained upon analysis of the protection rendered by **C** taurine, and **D** vitamin B12 against homocysteine-induced cytotoxicity. The results are expressed as mean ± SEM (n = 3)

To further understand the cytoprotective actions of taurine and vitamin B12, RAW 264.7 cells were treated with 0.9 mM H_2_O_2_, followed by treatment with the selected treatment doses to examine how the concentrations aid to mitigate the cytotoxic effects of H_2_O_2_. Based on the analysis of the results shown in Fig. 10[A, B], 50 mM taurine had a considerable cytoprotective impact when compared to higher dosages, but all of the selected vitamin B12 doses exhibited almost equal but significant cytoprotective potential. Similar to the cytoprotective experiment, the ability of taurine and vitamin B12 to mitigate the cytotoxic effects of 18 mM homocysteine on RAW 264.7 cells was investigated. The findings, as demonstrated in Fig. 10[C, D], show that taurine and homocysteine can significantly ameliorate the cytotoxic effects of high concentrations of homocysteine, with 50 mM taurine and 0.5 mM vitamin B12 showing the most significant results. However, even though all concentrations of taurine seem to have an overall ameliorative action against the cytotoxicity induced by homocysteine, vitamin B12 dose-dependently decreased in this ability with 3 mM vitamin B12 not with 3 mM vitamin B12 not being able to alleviate the cytotoxicity and instead further decreases the cell viability. All the analysis was further performed along with a 0.5 mM ascorbic acid as a standard.

#### Analysis of ROS scavenging potential of compounds

**Fig. 11:**
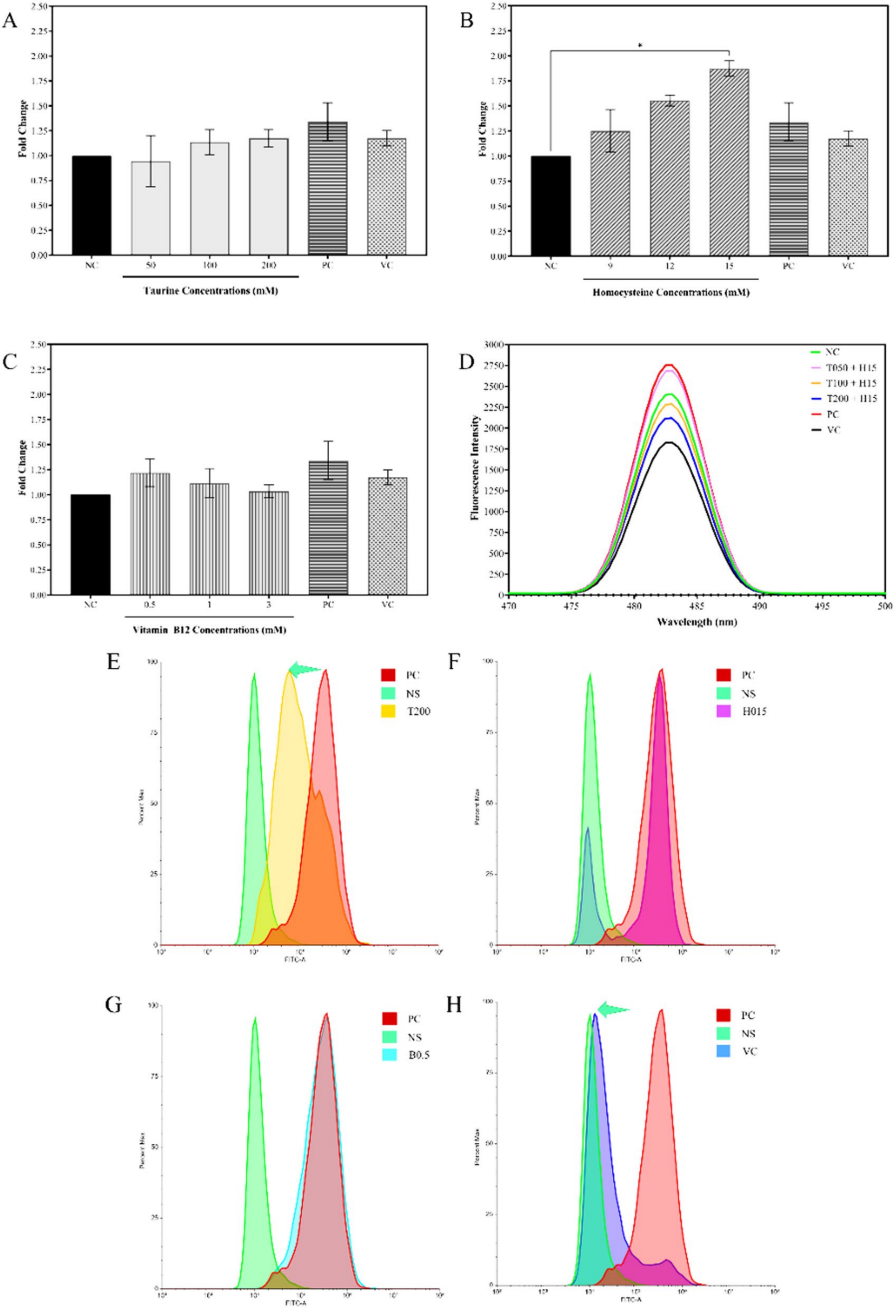
Representations of *in-vitro* assays performed on RAW 264.7 cells. The panels **A**–**D** Represents the ROS scavenging assay plots for **A** taurine, **B** homocysteine, **C** vitamin B12, and **D** taurine in combination with homocysteine. In the plots non-treated inactive macrophages are shown as negative control (NC) and the activated macrophages are shown as positive control (PC). The panels **E**–**H** represents the flow cytometric evaluation of the potential for ROS scavenging in LPS-stimulated RAW 264.7 cells for **E** 200 mM taurine, **F** 15 mM homocysteine, **G** 0.5 mM vitamin B12, and **H** 0.5 mM ascorbic acid. In the plots non-treated, unstained inactive macrophages are shown as negative control (NS) and the activated macrophages are shown as positive control (PC). The 0.5 mM ascorbic acid standard has been represented in the graphs as VC. The results are expressed as mean ± SEM (n = 3)

Following the cytoprotective and cytotoxicity protection studies, the compounds taurine, homocysteine, and vitamin B12 were investigated for their influence on ROS scavenging with the DCF-DA dye. Figure 11[A-C] shows that taurine in inactivated macrophages maintains the ROS at approximately the normal levels as observed relative to the control and the 0.5 mM ascorbic acid standard. However, homocysteine causes a dose-dependent increase in ROS formation within cells, with 15 mM homocysteine producing significantly more ROS than the control. Vitamin B12, on the other hand, had no appreciable ROS scavenging potential, and at a dosage of 0.5 mM, ROS levels were nearly comparable to the ascorbic acid standard. The same investigation, when combined with 15 mM homocysteine (Fig. 11[D]), revealed that the highest dose of 200 mM taurine had the greatest potential to scavenge ROS radicals forming within a cell as a result of homocysteine activity. It was observed that the ROS scavenging potential of taurine increased dose-dependently when in combination with 15 mM homocysteine. Similar to the aforementioned findings, the study performed in LPS—activated macrophages concluded that a 200 mM dosage of taurine may greatly reduce ROS generation within the cells, as seen in Fig. 11[E–H], comparable to 0.5 mM ascorbic acid, which serves as a reference. The ROS levels in activated macrophages treated with 15 mM homocysteine were found to be rather high, but the 0.5 mM dose of vitamin B12 did not appear to be effective in lowering ROS levels.

#### Analysis of nitric oxide scavenging potential of the compounds

**Fig. 12:**
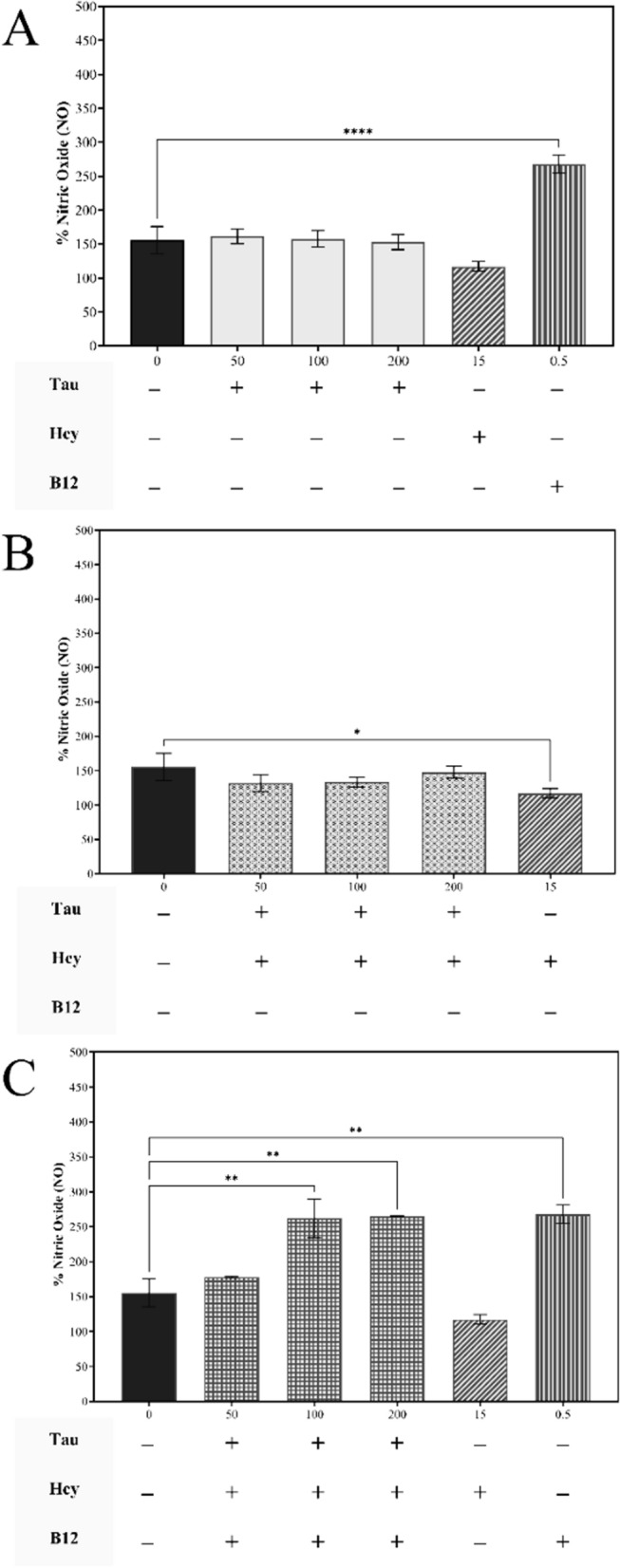
Analysis of *in-vitro* assays performed on differentiated Saos-2 cells. The panels **A**–**C** demonstrates the changes in NO (in percentage) levels in cells using **A** individual doses of taurine treatment doses in while also in **B** combination with 15 mM homocysteine, and **C** and in combination with both 15 mM homocysteine and 0.5 mM vitamin B12. The results are expressed as mean ± SEM (n = 3)

Following the experiments in RAW 264.7 cells, the compounds taurine, homocysteine, and vitamin B12 were studied for their effect on nitric oxide (NO) levels in osteoblast-like Saos-2 cells across a 7 day differentiation period with Griess' reagent. Figure 12 shows that all taurine treatment dosages maintained normal NO levels within the cells. In the same study, the 15 mM homocysteine dose was shown to reduce the nitrite levels significantly within the cells while the 0.5 mM vitamin B12 dose resulted in a significant rise in NO levels within cells. This was particularly evident in drug combination studies with the three taurine dosages combined with or without 0.5 mM vitamin B12. In the combination of taurine dosages with only 15 mM homocysteine, we notice that all of the doses manage to retain NO levels roughly equal to control levels thereby maintaining the normal nitrite levels within the cells. When in combination with 15 mM homocysteine and 0.5 mM vitamin B12, we find that only taurine doses 100 mM and 200 mM enhanced the NO levels significantly.

#### Analysis of mineralization potential of the compounds

**Fig. 13:**
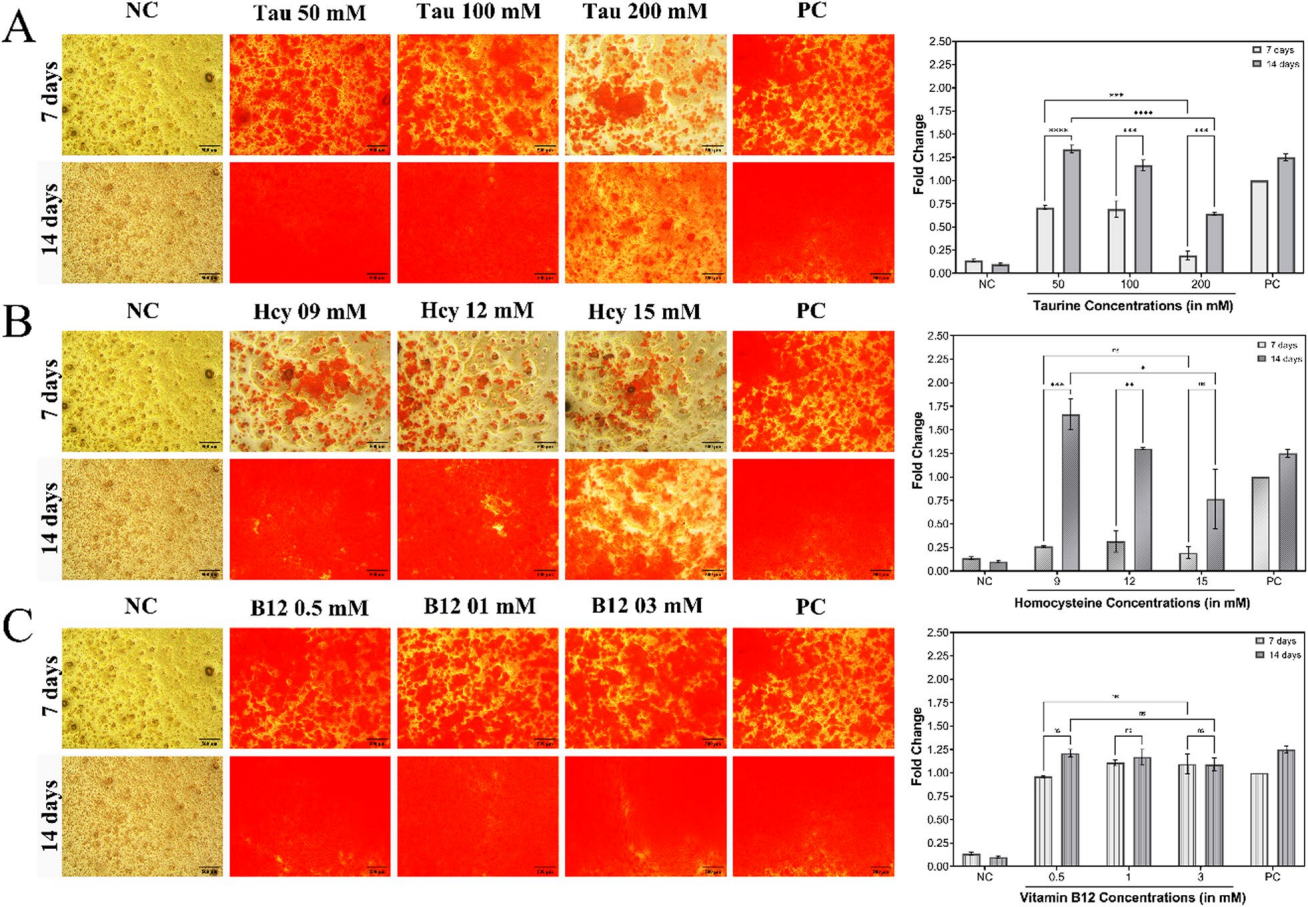
Analysis of *in-vitro* assays performed on differentiated Saos-2 cells. The panels **A**–**C** depicts the visualization and quantification of the amount of calcium deposition using **A** taurine treatment doses, **B** homocysteine treatment doses, and **C** vitamin B12 treatment doses. In the plots non-treated Saos-2 cells grown in normal complete media are shown as negative control (NC) and the Saos-2 cells that underwent differentiation in osteogenic media are shown as positive control (PC). The results are expressed as mean ± SEM (n = 3)

The amount of mineralization produced by taurine, homocysteine, and vitamin B12 over a period of 7 and 14 days was visualized and measured using the ALS assay, which was performed on Saos-2 cells that had been differentiated into osteoblast-like cells. Figure 13 illustrates that while all taurine treatment doses significantly increased the levels of mineralization by 14 days, 50 mM taurine was found to have caused the greatest increase. The 200 mM taurine dose demonstrated a significantly lower amount of calcium deposition than the other taurine doses under investigation. The results of the assays conducted in RAW 264.7 cells were also confirmed in Saos-2 cells through the unexpected observation that, although homocysteine did not exhibit much mineralization in all three treatment doses for the first 7 days, at the end of the 14 days, 9 mM and 12 mM homocysteine showed quite a significant rise in the calcium deposition. Although 15 mM homocysteine had subsequently enhanced the calcium deposition as well, the increase was not as significant as the previous seven days. On the other hand, the amount of mineralization caused by all the concentrations of vitamin B12 showed significantly higher levels of mineralization which was almost comparable after both 7 and 14 days. The 0.5 mM dose of vitamin B12 showed the highest mineralization in comparison to the rest of the doses after 14 days.

#### Gene expression analysis

**Fig. 14:**
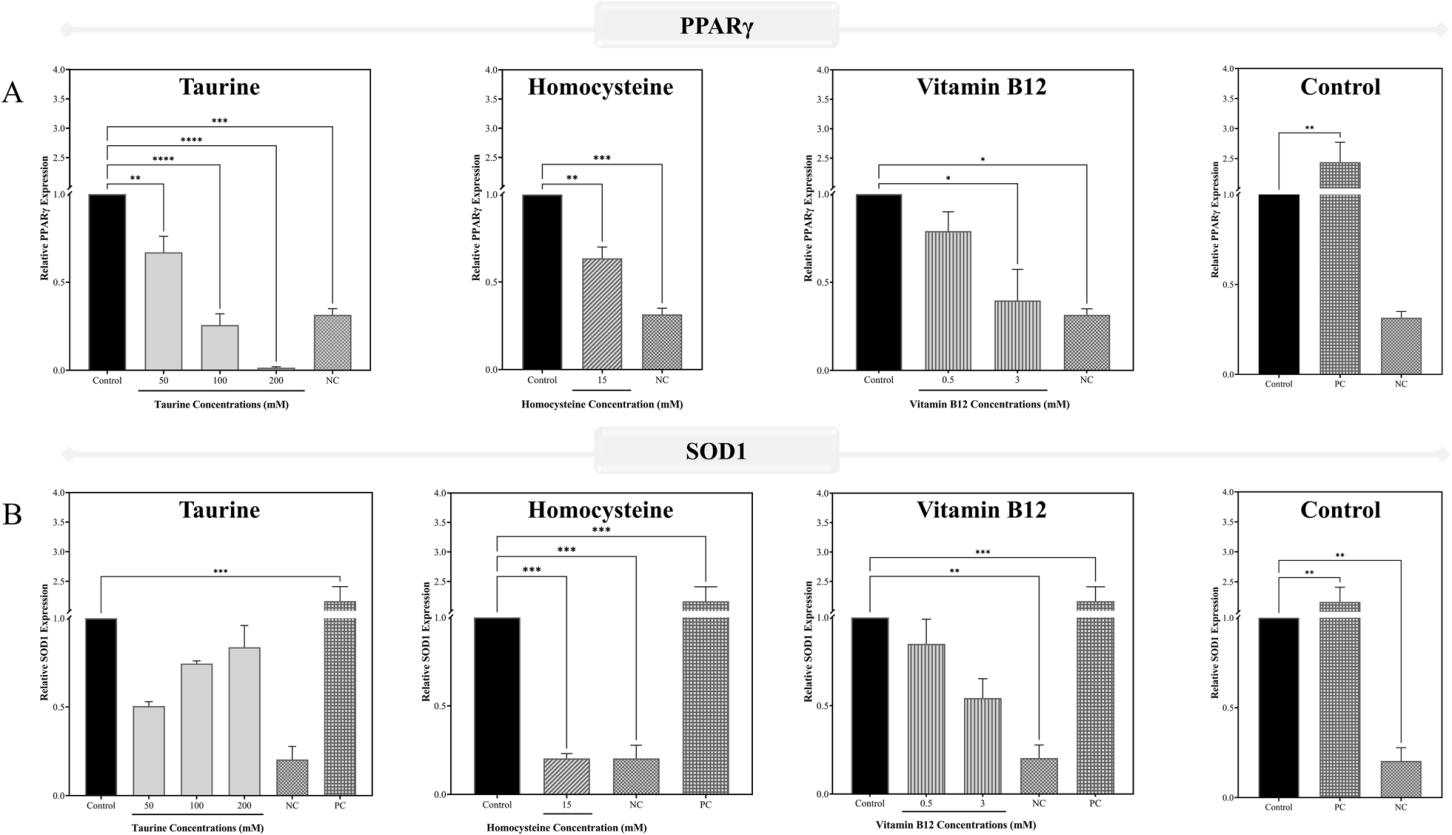
Validating *in-silico* observations using the mRNA expression of the genes of the prominent negative regulators of Wnt signaling pathway. The panels **A**, **B** represents the gene expression profiles for **A** PPARγ and **B** SOD1 in LPS-stimulated RAW 264.7 macrophages. In the plots antagonist of PPARγ (GW9662) are labelled as negative control (NC) and the agonist of PPARγ (pioglitazone hydrochloride) are shown as positive control (PC). The results are expressed as mean ± SEM (n = 3)

To validate the results of the *in-silico* investigation and corroborate the results of the *in-vitro* testing, gene expression for the negative regulators of the Wnt signaling pathway was performed. The findings of gene expression are shown in Fig. 9. PPARγ and SOD1 gene expression was measured in activated RAW 264.7 macrophages treated with taurine (50, 100, and 200 mM), homocysteine (15 mM), and vitamin B12 (0.5 and 3 mM). The study compared them to the PPARγ agonist pioglitazone hydrochloride (50 µM) and antagonist GW9662 (10 µM) as positive and negative controls, respectively. Figure 14[A] shows that taurine and vitamin B12 have a dose-dependent impact on downregulating PPARγ. However, as shown in Fig. 14[B], taurine was found to dose-dependently increase the expression levels of SOD1. The most significant effects for taurine were seen at 200 mM taurine. Homocysteine was observed to reduce PPARγ and SOD1 levels. Taurine and homocysteine were shown to downregulate PPARγ, with taurine having quite significant effect, as predicted from the *in-silico* investigations involving taurine and homocysteine in this study. Based on these results, a 200 mM and 0.5 mM individual doses of taurine and vitamin B12 respectively were chosen for the remaining genetic investigations in differentiated osteoblast-like cells such as Saos-2, as well as homocysteine and RANKL-induced osteoclasts from RAW 264.7 cells.

**Fig. 15:**
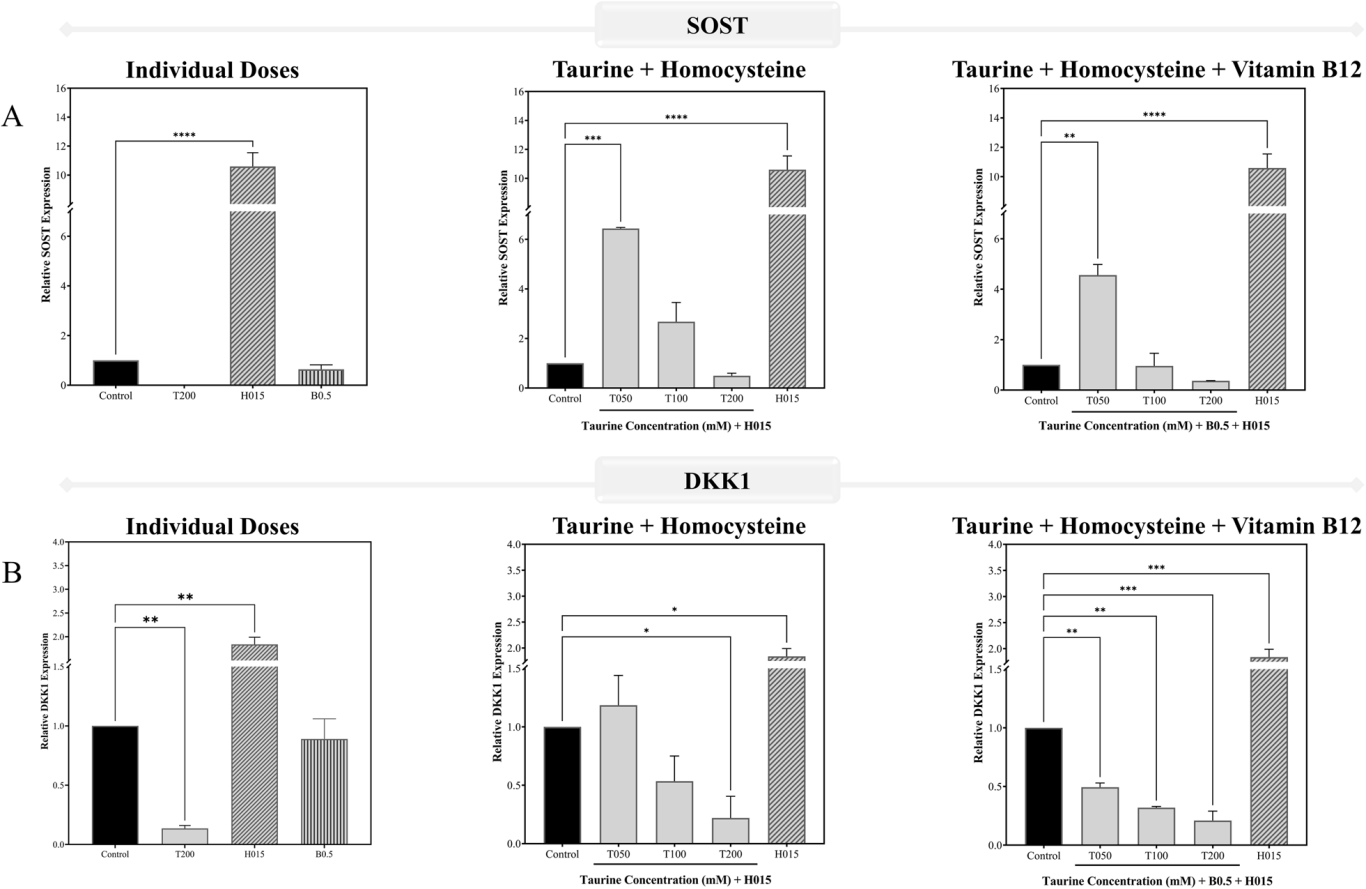
Validating *in-silico* observations using the mRNA expression of the genes of the prominent negative regulators of Wnt signaling pathway. The panels **A**, **B** represents the gene expression profiles for **A** SOST and **B** DKK1 in differentiated osteoblast-like Saos-2. The results are expressed as mean ± SEM (n = 3)

Figure 15[A-B] illustrates the genetic assessments performed on differentiated osteoblast-like cells where the individual dose of 200 mM taurine almost suppressed the expression of SOST and significantly downregulated the DKK1 levels, both of which have been reported to be influenced by PPARγ levels within the cells. To better understand the role of taurine by itself and in combination with vitamin B12 in the presence of high levels of homocysteine in the blood, combination studies were conducted, all of which point to the fact that 200 mM taurine may be the best for significantly downregulating the most important negative regulators of the Wnt signaling pathway in SOST and DKK1. Homocysteine was shown to significantly increase the levels of these negative regulators, which were significantly lowered by taurine treatment dosages, with or without vitamin B12. However, the synergistic impact of vitamin B12 and taurine resulted in significantly higher downregulation of SOST and DKK1 expression levels. Individually, vitamin B12 (0.5 mM dose) failed to have a significant impact on the genes, however a modest drop in expression level was noted.

**Fig. 16:**
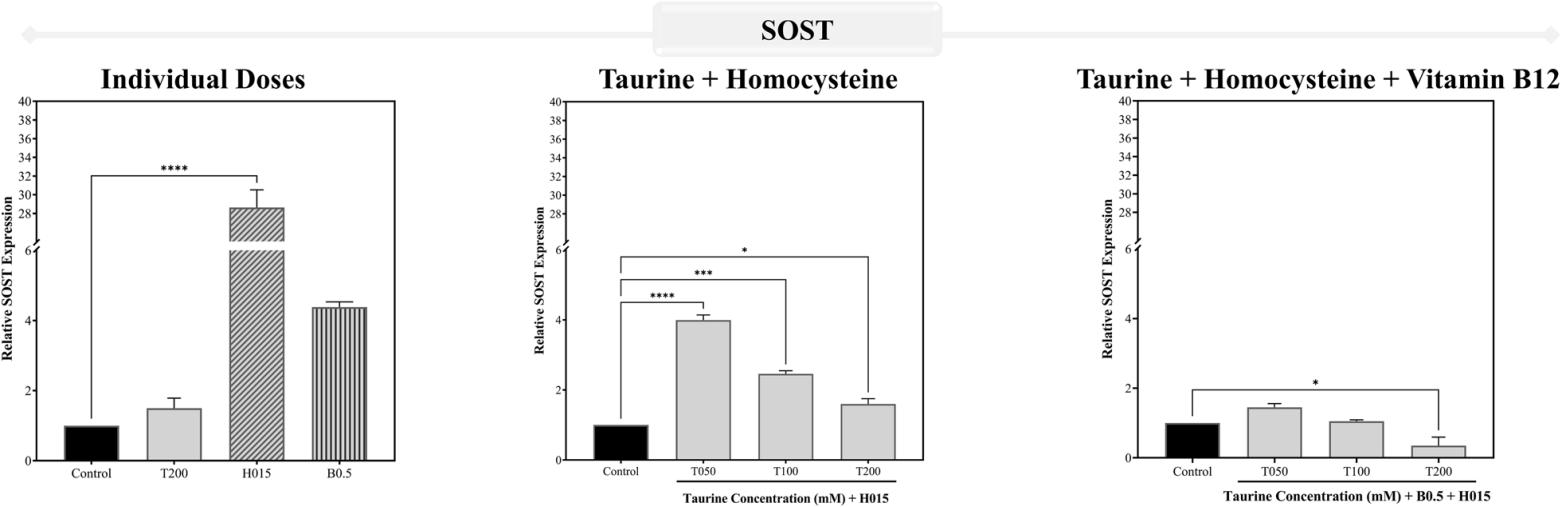
Validating *in-silico* observations using the mRNA expression of the genes of the prominent negative regulators of Wnt signaling pathway. The plot represents the SOST expression profile for RANKL-induced osteoclast added along with homocysteine from RAW 264.7 cells. The results are expressed as mean ± SEM (n = 3)

Osteoclasts induced by RANKL and homocysteine from RAW 264.7 macrophages revealed considerable elevation of SOST levels, as seen in Fig. 16. Similar to the osteoblast-like cells, 200 mM taurine dosage significantly decreased SOST levels in the osteoclasts individually as well as in combination vitamin B12. The osteoclast differentiation was validated by checking the changes in its morphology prior to this using the May-Grünwald Giemsa staining (Fig. S6).

### Analogue study analysis

A total of 141 taurine analogues were obtained from the PubChem database using a Tanimoto threshold of 90%. From these, 72 conjugated taurine analogues were selected for further analysis and docked with the homology-modeled protein to identify analogs with improved binding interactions and activity compared to taurine. Docking conditions for the analogs were consistent with those used for taurine, focusing exclusively on the ligand-binding domain (LBD) of the target protein. The top ten analogs, ranked by docking energy, are summarized in Table 4, which also includes their docking scores and inhibition constants. The highest-ranked compound, TA-64, demonstrated a binding free energy of −5.4 kcal/mol.

**Table 4:** The table demonstrates the molecular docking results

Analogue codes	SMILES	Binding affinity (kcal/mol)	Inhibition constant (mM)
TA-16	C(C(S(= O)(= O)O)S(= O)(= O)O)N	−5.2	0.22
TA-39	C(C(N)N)S(= O)(= O)O	−4.6	0.57
TA-45	C(C(N)S(O)(O)O)S(= O)(= O)O	−4.8	0.41
TA-47	C(CS(= O)(= O)O)NS(= O)(= O)O	−4.9	0.35
TA-50	C(C(N)S(= O)(= O)O)S(= O)(= O)O	−5.3	0.18
TA-52	C(CS(= O)(= O)O)NS(= O)[O-]	−4.7	0.49
TA-53	C(CS(= O)(= O)O)NI	−4.6	0.57
TA-64	C(CS(= O)(= O)O)NCl(= O) = O	−5.4	0.16
TA-65	C(C(S(= O)(= O)O)S(= O)(= O)Cl)N	−4.8	0.41
TA-70	C([C@@H](N)S(= O)(= O)O)S	−4.8	0.41

The binding affinity and inhibition constant for the top 10 docked taurine analogues with PPARγ have displayed in the table

The absorption, distribution, metabolism, and excretion (ADME) properties of the top ten taurine analogs, selected based on their docking scores with PPARγ, were predicted using the SwissADME web server [54]. This tool offers prediction accuracy ranging from 72 to 94%. ADME analysis evaluates the physicochemical and biological properties of the compounds, alongside their drug-likeness, providing crucial insights into their potential efficacy and suitability for in vivo applications. Table S2 demonstrates the screening process, during which compounds TA-50, TA-45, and TA-16 were excluded from further analysis. Subsequently the remaining 7 taurine analogues were screened for toxicity using the ProTox-II web server [55]. The web server could not predict the toxicity data for the taurine analogue TA-64 and hence, it was excluded from the study. The screening process for toxicity has been recorded in Table S3. The six remaining analogues were identified as having potential antagonistic modulatory effects on the PPARγ protein and their specific interactions have been depicted in Figure S7.

## Discussion

The role of PPARγ in regard to osteogenesis is quite complex as seen from previous literature with PPARγ activation being associated with the increase of negative regulators of the Wnt signaling pathway such as sclerostin (SOST) [23] and Dickkopf-related protein 1 (DKK1) [56]. The activation of PPARγ leads to adipogenesis and thereby a subsequent increase in osteoclastogenesis after the osteoblasts start to undergo apoptosis making its inactivation crucial for the process of bone formation to occur [21]. In this work, PPARγ inhibition was thus identified as a suitable strategy for improving bone formation. Given the conflicting reports regarding homocysteine’s influence on PPARγ [7–9], we sought to clarify its effects on osteoporosis in this study, while also examining the contrasting effects of taurine. The results from the study shows conclusively that taurine and homocysteine both induce inhibition of PPARγ by binding and modulating the important helices H3, H4/5, H11 and H12 in an *in-silico* setting with both the compounds binding and inhabiting different grooves within the PPARγ LBD. Given that both are amino acids that have smaller molecular weights and sizes, it is reasonable to expect that they would interact with sites where their effects are more pronounced compared to the typical agonists and antagonists linked to PPARγ. However since both the compounds showed inhibition towards PPARγ, it was essential to conduct *in-vitro* tests to elucidate their mechanisms of action concerning osteoporosis.

Molecular docking and dynamics provided critical insights into the interactions between taurine, homocysteine, and PPARγ. Taurine exhibited a stronger binding affinity (−4.1 kcal/mol) and formed three hydrogen bonds with PPARγ, whereas homocysteine had a slightly lower binding affinity (−4.0 kcal/mol), forming a salt bridge instead of hydrogen bonds. These findings suggested that taurine may have a more stable and effective interaction with the receptor. Both ligands interacted with important helices in the PPARγ ligand-binding domain (LBD), particularly H3, H4/5, H11, and H12, which are common targets for PPARγ inhibitors.

The molecular dynamic simulations reveal key insights into the stability and dynamics of PPARγ when bound to homocysteine and taurine. Homocysteine exhibits greater stabilizing effects on the receptor, as seen through lower RMSD, RMSF, and enhanced compactness, suggesting its potential as a reliable modulator of PPARγ activity. In contrast, taurine induced significant conformational shifts, particularly in the activation helix H12, and maintains a higher number of intermolecular hydrogen bonds, indicating a more dynamic and destabilizing interaction with the ligand-binding domain. The interaction between taurine and residues such as 224F, 228Q, and 285E seems to drive this destabilization. Homocysteine, conversely, preserves the structural integrity of the receptor with few alterations, especially in the activation helix H12, highlighting its overall stabilizing function. Its potential modulation is rendered chiefly through the helices H4/5 and H11. When both ligands are simulated simultaneously, taurine's destabilizing effect prevails, inducing perturbations in critical helices H3 and H12, even in the presence of homocysteine. This dynamic interaction suggests that taurine may influence PPARγ differently than classical agonists or antagonists, underscoring its value for further investigation in drug development.

The subsequent *in-vitro* study explored the cytoprotective, antioxidant, and osteogenic potential of taurine, homocysteine, and vitamin B12, focusing on their relevance in osteoporosis treatment through various assays. The cytotoxicity assays revealed that taurine exhibited strong protection against H_2_O_2_ and homocysteine-induced damage in RAW 264.7 cells, especially at 50 mM, while vitamin B12 demonstrated a dose-dependent decrease in cytoprotective potential, with higher concentrations failing to mitigate the cytotoxic effects of homocysteine. These findings suggest the importance of taurine in protecting bone cells from oxidative damage, which is relevant to osteoporosis, where oxidative stress contributes to bone resorption [57, 58]. The ROS scavenging assays showed that taurine effectively reduced ROS levels in both inactive and LPS-activated macrophages, suggesting its potential role in protecting bone cells from oxidative stress, which is associated with increased bone loss in osteoporosis. In contrast, homocysteine significantly increased ROS levels in a dose-dependent manner, exacerbating oxidative damage, while vitamin B12 showed limited ROS scavenging ability. Taurine’s ROS-scavenging effects were especially notable when combined with homocysteine, indicating its ability to counteract homocysteine-induced oxidative stress.

The nitric oxide (NO) assays were conducted in differentiating Saos-2 cells due to the known role of NO in promoting bone formation [59]. Increased NO levels are associated with enhanced osteoblastic activity and mineralization, critical factors in osteoporosis management. Taurine maintained normal NO levels in the differentiating Saos-2 osteoblast-like cells, indicating its positive role in promoting bone health. Interestingly, homocysteine reduced NO levels, which could impair bone formation, while vitamin B12 increased NO levels, suggesting its potential to stimulate osteoblast activity. Combination studies revealed that taurine, particularly at higher doses, helped to restore normal NO levels even in the presence of homocysteine, thereby facilitating bone formation. The combination of taurine and vitamin B12 was found to be instrumental in increasing the NO levels within the cells, further consolidating its synergistic potential in osteogenesis.

The mineralization assays further confirmed taurine’s ability to enhance bone formation, as all taurine doses significantly increased calcium deposition in Saos-2 cells by day 14, with 50 mM taurine showing the highest effect. The decrease in the mineralization upon the increase in doses for taurine could be attributed to the probable anti-cancer properties of taurine [60, 61]. Homocysteine exhibited a delayed but significant increase in mineralization, particularly at lower doses with the 15 mM dose being detrimental towards proper mineralization in osteoblasts, while vitamin B12 promoted consistent mineralization across all concentrations. The explanation for the abrupt rise in homocysteine mineralization between the seventh and fourteenth days is unknown to us at this moment and warrants for further investigations. These findings are crucial in osteoporosis, where enhancing bone mineralization is vital to counteract bone loss. All of these findings support taurine's position as an osteogenesis promoter, either alone or in conjunction with vitamin B12, whereas the 15 mM dosage of homocysteine has been identified as the most potent hyperhomocysteinemic dose that causes osteoporotic conditions within cells.

The gene expression analysis examined the effects of taurine and vitamin B12 on the expression of PPARγ and SOD1 genes in macrophages in addition to providing critical insights into their osteogenic potential, particularly their impact on negative regulators of the Wnt signaling pathway, such as SOST and DKK1, which play vital roles in bone formation and remodeling. The genes PPARγ and SOD1 were selected for analysis in activated RAW 264.7 macrophages due to their central roles in inflammation, oxidative stress, and bone remodeling. As discussed before, PPARγ is a key regulator of adipogenesis and inhibits osteoblast differentiation, making it a critical target in the macrophage-driven inflammatory environment, where it contributes to bone resorption and osteoclastogenesis. Conversely, SOD1 plays a vital role in protecting cells from oxidative damage by scavenging ROS, an important factor in macrophage-induced bone degradation through subsequent osteoclast formation. By evaluating these genes, the study aimed to assess taurine's and vitamin B12's ability to reduce inflammatory damage and oxidative stress in macrophages, ultimately impacting the differentiation of macrophages towards mature osteoclasts thereby being detrimental towards bone health. In typical studies, PPARγ and SOD1 exhibit a positive correlation, with PPARγ often acting as a regulatory factor that induces the expression of SOD1 in certain contexts [62, 63]. PPARγ activation can enhance SOD1 expression to mitigate oxidative stress, as both genes are involved in metabolic and oxidative stress pathways. This coordination aids in adipogenesis and the management of cellular oxidative conditions. However, the study with taurine in activated macrophages showed an inverse correlation between these two genes, particularly at the 200 mM dose. As taurine significantly downregulated PPARγ, it concurrently upregulated SOD1, indicating that taurine could be enhancing SOD1 expression through a PPARγ-independent mechanism. This suggests that taurine's effect on SOD1 is potentially mediated via alternative pathways, such as those involved in cellular stress responses, like the NRF2 pathway or other oxidative stress-related signaling routes [64], bypassing PPARγ's usual influence. In contrast, vitamin B12 did not show the same inverse correlation, and its ability to influence SOD1 expression seemed to be more aligned with PPARγ's regulation. This implies that vitamin B12 may rely more on traditional PPARγ-dependent pathways to influence oxidative stress management, rather than activating alternative signaling mechanisms as taurine does. Thus, while SOD1 can seemingly be regulated through both PPARγ-dependent and PPARγ-independent pathways, taurine's distinct capacity to dissociate SOD1 expression from PPARγ suppression highlights its unique potential in reducing oxidative stress and promoting bone health through multiple cellular pathways, an effect that might not be as pronounced with vitamin B12. This dual action reported in the study contributes to the understanding of potential therapeutic effect of taurine in conditions like osteoporosis.

The study also supported prior findings [8, 9, 65] that homocysteine significantly suppressed PPARγ expression, consistent with our *in-silico* findings. Based on our findings, we infer that when homocysteine binds to PPARγ, it acts as a probable antagonist. PPARγ inactivation should, in principle, stimulate osteoblastogenesis while inhibiting adipogenesis. However, in order to understand the reason for the dramatic increase in the unregulated ROS levels in cells ultimately leading towards osteoclastogenesis, it is to be considered that homocysteine contains a 'thiol' (R-SH) group that undergoes auto-oxidation in the presence of transition metals and molecular oxygen, resulting in the production of ROS [66, 67]. Homocysteine's pro-oxidant nature causes the generation of multiple oxidants, including hydrogen peroxide and superoxide anion, which may generate the potent oxidant peroxynitrite [68]. Thus, in an environment such as hyperhomocysteinemia, where the level of homocysteine is disproportionally higher than normal, in addition to binding to all available PPARγ jeopardizing the normal ROS scavenging mechanisms, there is still enough homocysteine in the systemic circulation to promote oxidizing conditions within the cells due to the amount of auto-oxidation of homocysteine thiol groups. This in addition to downregulating the SOD1 gene, creates an overall oxidizing environment, which promotes the halting of osteogenesis and the onset of osteoclastogenesis. Furthermore, homocysteine produces ROS via mitochondrial stress and its agonistic action on NMDA-R, resulting in a massive intracellular calcium influx [69] and, eventually, a surge in matrix metalloproteinases (MMPs) [7].

Following the analysis of PPARγ and SOD1 in macrophages, the study shifted focus to the negative regulators of the Wnt signaling pathway – SOST and DKK1, both of which inhibit osteoblast differentiation and promote osteoclastogenesis, leading to bone loss. The study investigated taurine and vitamin B12's ability to downregulate these genes to enhance bone formation and reduce bone resorption. The 200 mM dose of taurine was recognized as the potent individual treatment dose moving forward and was found to significantly downregulate SOST and DKK1, thereby promoting osteoblast activity and reducing osteoclast activity, which is critical for healthy bone remodeling. The selected dose of 0.5 mM vitamin B12 based on the results observed from assays and expression of the PPARγ and SOD1 genes in macrophages, when combined with taurine, further amplified this downregulation, demonstrating a synergistic effect in supporting bone formation and mitigating resorption. Homocysteine, in contrast, upregulated SOST and DKK1, exacerbating the inhibition of the Wnt signaling pathway and promoting bone loss. This underscores the detrimental role of hyperhomocysteinemia in osteoporosis and further highlights taurine's potential therapeutic efficacy in counteracting these effects individually and in combination with vitamin B12.

In the homocysteine and RANKL-induced osteoclast model, SOST was the focus because it directly suppresses osteoblast activity and promotes osteoclastogenesis. By studying SOST in osteoclasts derived from RAW 264.7 macrophages, the research aimed to explore taurine's effect on mitigating excessive bone resorption, particularly in a pro-osteoclastogenic environment influenced by hyperhomocysteinemia. The same pattern of downregulation of SOST was seen for the hyperhomocysteinemia induced osteoclast model for taurine individually and in combination with vitamin B12 with the combination dose of 200 mM taurine and 0.5 mM vitamin B12 showing the most efficacy in alleviating the osteoporotic conditions rendered by excessive homocysteine accumulation within the cells. Overall, taurine, especially at higher doses, exhibits significant potential for osteoporosis management by modulating key genetic pathways by reducing PPARγ and increasing SOD1 to protect against oxidative stress, and downregulating SOST and DKK1 to promote bone formation and simultaneously inhibit bone resorption. Combined with vitamin B12, this effect is enhanced, suggesting that taurine and vitamin B12 together may offer a powerful therapeutic strategy for protecting against hyperhomocysteinemia induced osteoporosis and restoring bone health.

**Fig. 17:**
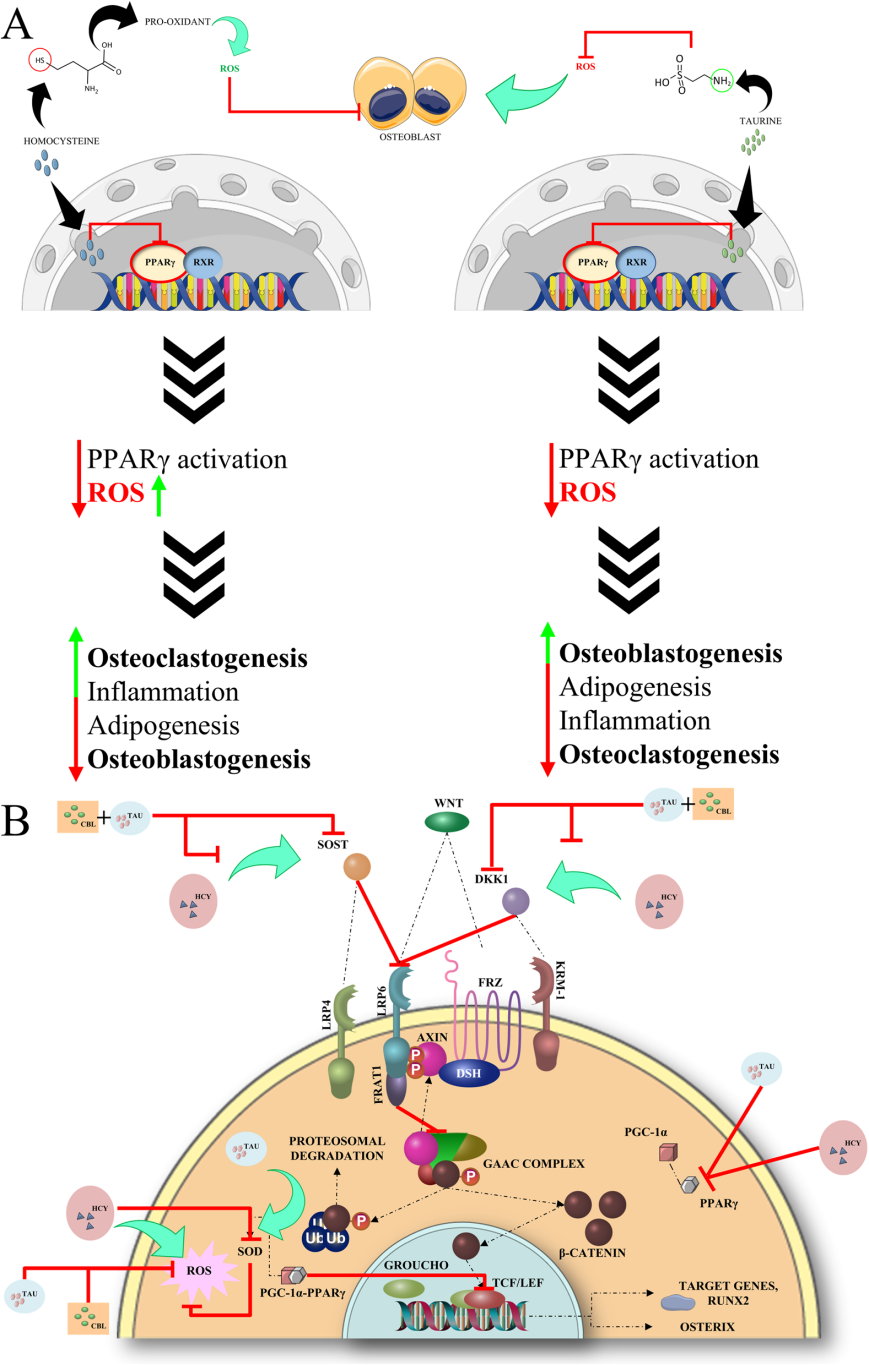
A schematic representation of the mechanism of action of taurine and homocysteine on the negative regulators of Wnt signaling. The panel **A** represents the mechanism of action of homocysteine and taurine on PPARγ alongwith the overview of the effects bestowed by their actions, and **B** shows the action of both homocysteine and taurine individually and in combination with vitamin B12 on the negative regulators of the Wnt signaling pathway as a detail snapshot

So, the mechanism by which osteoporotic conditions develop in hyperhomocysteinemic patients is most likely due to both PPARγ inactivation and thiol auto-oxidation in order to promote ROS levels within the cells, with this cascade creating a favorable environment for the production of SOST in a PPARγ independent manner. Taurine, as an antioxidant, relieves much of the oxidative stress caused by homocysteine in cells by reacting with oxidants such as hypochlorous acid (HOCl) to form taurine chloramine, which acts as an anti-inflammatory agent, detoxifying and alleviating oxidative stress caused by excessive surge of nitric oxide (NO), hydrogen peroxide (H_2_O_2_), and hydroxyl (OH-) ions. It also protects sulfhydryl (-SH) groups indirectly by protecting glutathione (GSH) levels [70], which in turn protects non-protein -SH groups such as those found in homocysteine from oxidation, in addition to upregulating ROS scavenging genes such as SOD1. As a result, the ROS levels that may be produced in situations of hyperhomocysteinemia are significantly reduced. This in turn directly and/or indirectly affects the Wnt signaling pathway negative regulators such as SOST and DKK1 thereby promoting osteogenesis as predicted in previous studies [24]. Figure 17 shows a detailed illustration of the inferences drawn from the study.

In this study, we also aimed to identify taurine analogues with enhanced pharmacological properties to overcome the limitations associated with amino acids, potentially increasing their efficacy and action potential as drugs. Taurine analogues retrieved from the PubChem database were subjected to molecular docking against the PPARγ receptor, following the same methodology discussed previously. Post-docking analysis revealed that the top ten poses exhibited significant affinity towards the PPARγ receptor, with the core structure of the analogues still interacting with the PPARγ LBD. These top ten taurine analogues (Table 4) were further screened for their ADMET and other key pharmacokinetic properties (Tables S2 and S3). As a result of this screening, four analogues – TA-50, TA-45, and TA-16 – were eliminated due to poor absorption in the gastrointestinal tract while TA-64 was excluded due to lack of predicted and experimental data.

## Conclusion

In this study, taurine demonstrated significant potential for osteoporosis management by modulating key genetic pathways, particularly through the inhibition of PPARγ. *In-silico* analysis predicted the role of taurine and homocysteine as probable antagonists of PPARγ through distinct binding interactions, influencing osteogenic processes. The involvement of the β-sheets suggests a probable inverse agonist role for taurine. *In-vitro* assays further validated these findings, where taurine, especially at 200 mM, downregulated PPARγ and upregulated SOD1, highlighting a unique PPARγ-independent mechanism for enhancing oxidative stress defense. Taurine also downregulated negative regulators of the Wnt signaling pathway, SOST and DKK1, in osteoblasts, promoting bone formation while simultaneously reducing bone resorption. Combined with vitamin B12, taurine’s osteogenic potential was amplified, presenting a promising strategy for combating hyperhomocysteinemia induced osteoporosis. Moreover, the study screened and identified six potential analogues of taurine with possibly better pharmacological and biologically activity.

However, it is important to acknowledge the limitations of this study. While computational models, such as molecular docking and dynamic simulations, provide valuable insights into ligand-receptor interactions, they are inherently predictive and may not fully capture the complexities of biological systems. Therefore, we recognize that extensive *in-vitro* validation, surpassing the scope of our current experiments, will be required to confirm these computational predictions and establish a more comprehensive understanding of taurine and homocysteine’s effects on PPARγ. Additionally, we acknowledge that we utilized secondary cell lines in this study rather than primary cells. While secondary cell lines may not fully replicate the physiological conditions found in primary cells or tissues, they are widely used in preclinical research due to their reproducibility, ease of handling, and well-established characteristics. Importantly, our experimental results are still robust and provide valuable insights into the potential mechanisms underlying taurine and vitamin B12’s effects on osteoporosis. However, we recognize that secondary cell lines, being immortalized, may exhibit minor differences in their response compared to primary cells. Furthermore, given the potential anti-cancer properties of taurine, the behavior of secondary cell lines may be influenced by certain factors. This may not fully mirror the more nuanced interactions occurring in primary osteoblasts and osteoclasts. Despite this, the consistency of our findings across different assays and the clear trends observed in our data strongly support the validity of our conclusions. Nevertheless, future studies should aim to validate these findings in primary cell cultures to better understand the physiological relevance of our results and to further explore the potential therapeutic applications of taurine and vitamin B12 in osteoporosis.

Our present work provides a foundational basis for further research, but future perspectives should focus on exploring the long-term effects of taurine and vitamin B12 in animal models of osteoporosis, elucidating their molecular mechanisms across different cellular pathways, and evaluating their potential clinical applications.

## Supplementary Information

Supplementary Material 1.

## Data Availability

All relevant data are within the paper. The raw data used and/or analyzed during the current study are available from the corresponding author upon reasonable request.
