# Supplementary material for: Integrative in-silico and in-vitro analysis of taurine and vitamin B12 in modulating PPARγ and Wnt signaling in hyperhomocysteinemia-induced osteoporosis
Source: Biol Direct. 2024 Dec 20;19:141. doi: 10.1186/s13062-024-00581-z (PMC11662456; doi:10.1186/s13062-024-00581-z)
Supplement: Supplementary file 1 — Supplementary Material 1. [file 13062_2024_581_MOESM1_ESM.docx]

Integrative *in-silico* and *in-vitro* analysis of taurine and vitamin B12 in modulating PPARγ and Wnt signaling in hyperhomocysteinemia-induced osteoporosis

**Supplementary Data**


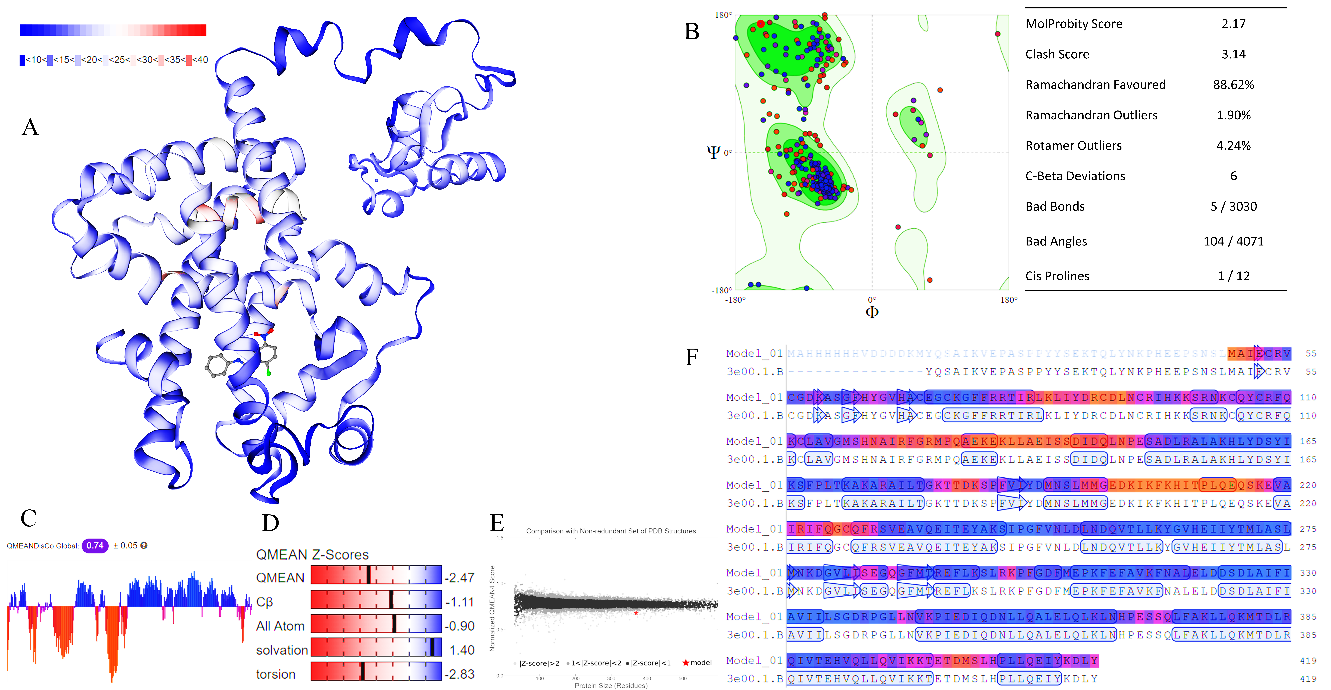


**Figure S1.** **Homology modeled structure validation for PPARγ.** **[A]** PPARγ homology modeled structure based on the B-factor scores with GW9 ligand bound to the ligand binding site and two zinc ions bound at the DNA binding domain; **[B]** The Ramachandran plot and statistics obtained upon homology modelling; **[C]** QMEAN per-residue plot and score; **[D]** The QMEAN Z-score analysis **[E]** Comparison for validating the quality of the model in respect to high resolution crystal structures available in PDB; **[F]** The model-template alignment as per the hydrophobicity residue schemes.


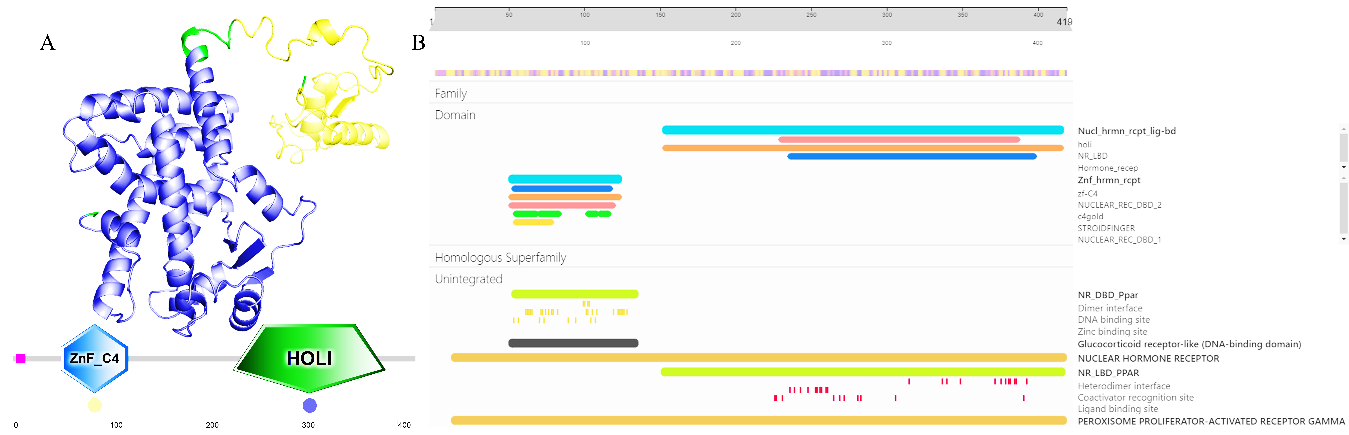


**Figure S2.** **Recognition of functional domains.** **[A]** The zinc finger, DNA binding domain and the ligand binding domain colour coded and shown through the SMART graphical representations of the search made for recognizing the domains present in the homology modelled protein; **[B]** InterPro scan of the homology modelled protein confirming the presence of those domains using various other databases alongwith the identified ligand binding residues, coactivator recognition residues and heterodimer interface.


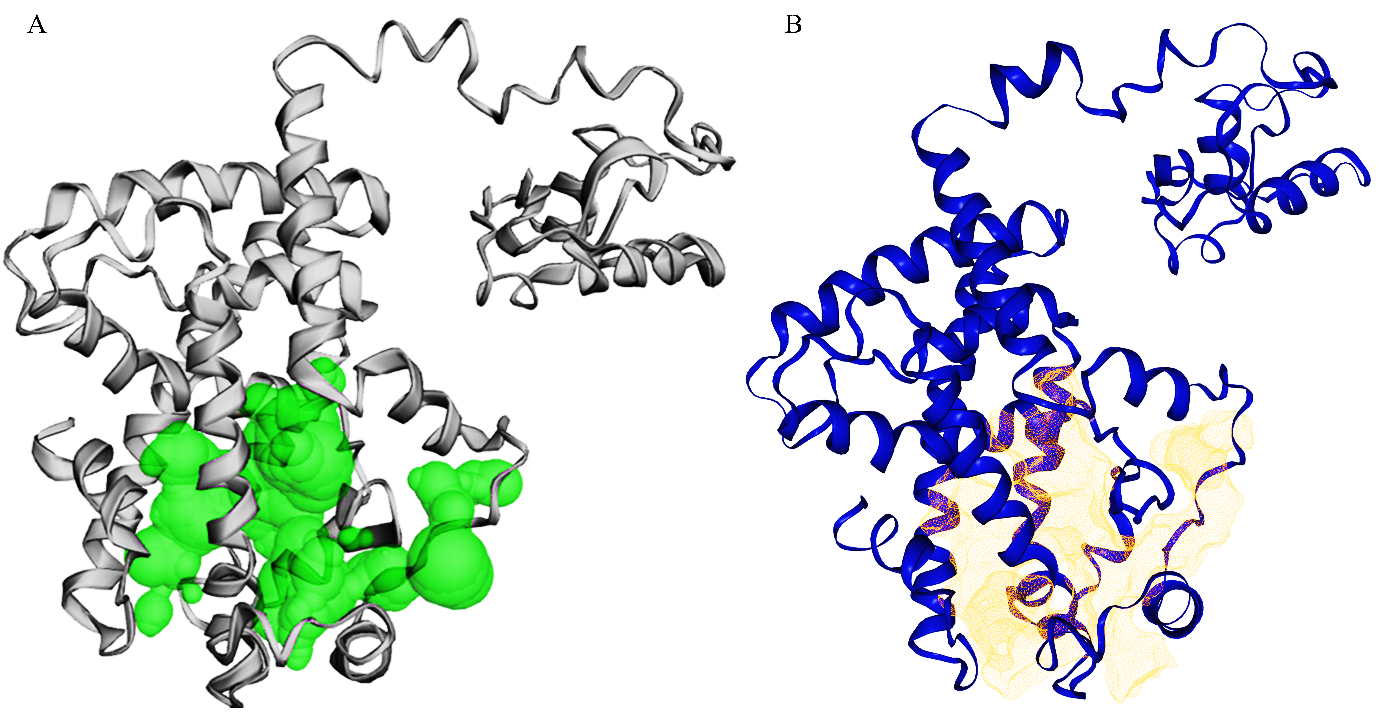


**Figure S3.** **Binding site identification.** The binding sites were predicted and identified using **[A]** CASTp server **[B]** Protein Plus server.


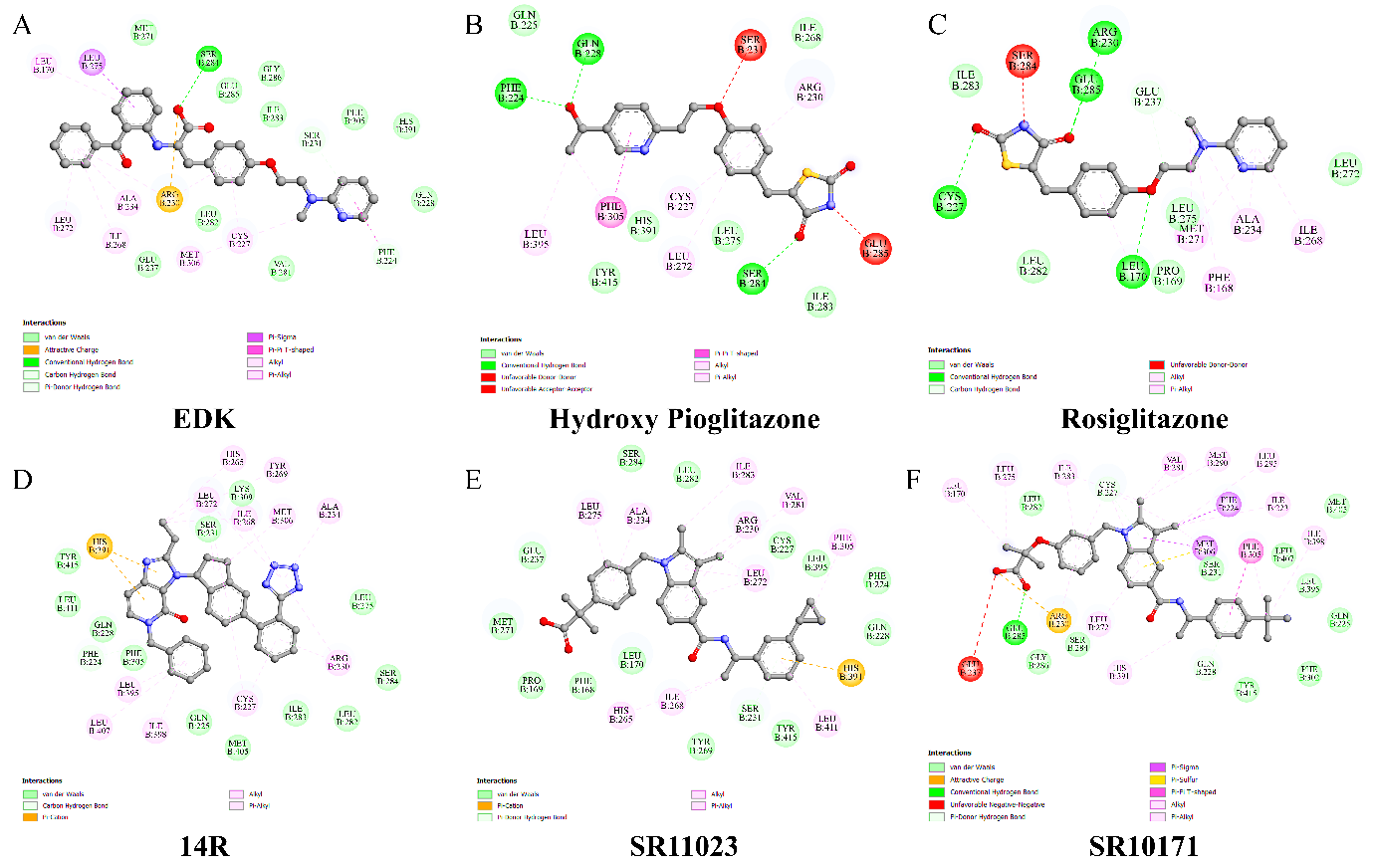


**Figure S4. Molecular docking interaction diagram with agonists and antagonists of PPARγ to validate the binding pocket and important helices.** The panels **[A-C]** show the 2-D interactions between agonists for the target receptor, **[D-E]** demonstrates the 2-D interactions between antagonists and the target receptor, and **[F]** denotes the 2-D interactions between inverse agonist and the target receptor.

**Table S1. The table depicts the binding affinity and residue interactions of the compounds of interest with the target.** The interacting residues have also been listed in the table.

| ***Name*** | ***Binding Affinity (kcal/mol)*** | ***Hydrogen Bond Interactions*** | ***Hydrophobic Interactions*** | ***Salt Bridges Interactions*** |
| --- | --- | --- | --- | --- |
| **Agonists** | | | | |
| ***EDK*** | -9.4 | 284S | 170L, 224F, 227C, 230R, 231S, 234A, 268I, 272L, 275L, 306M | ** |
| ***Hydroxy pioglitazone*** | -8.8 | 224F, 228Q, 284S | 227C. 230R, 231S, 272L, 286E, 305F, 395L, | ** |
| ***Rosiglitazone*** | -8.1 | 170L, 227C, 230R, 285E | 168F, 234A, 268I, 271M, 284S | ** |
| **Antagonists** | | | | |
| ***14R*** | -10.8 | ** | 224F, 227C, 230R, 234A, 265H, 268I, 269Y, 272L, 306M, 395L, 398I, 407L | ** |
| ***SR11023*** | -11.1 | ** | 230R, 231S, 234A, 265H, 268I, 272L, 275L, 281V, 283I, 305F, 391H, 411L | ** |
| **Inverse Agonist** | | | | |
| ***SR10171*** | -11 | 285E | 170L, 223I, 224F, 227C, 228Q, 230R, 237E, 275L, 281V, 283I, 290M, 295L, 305F, 306M, 391H, 398I | ** |


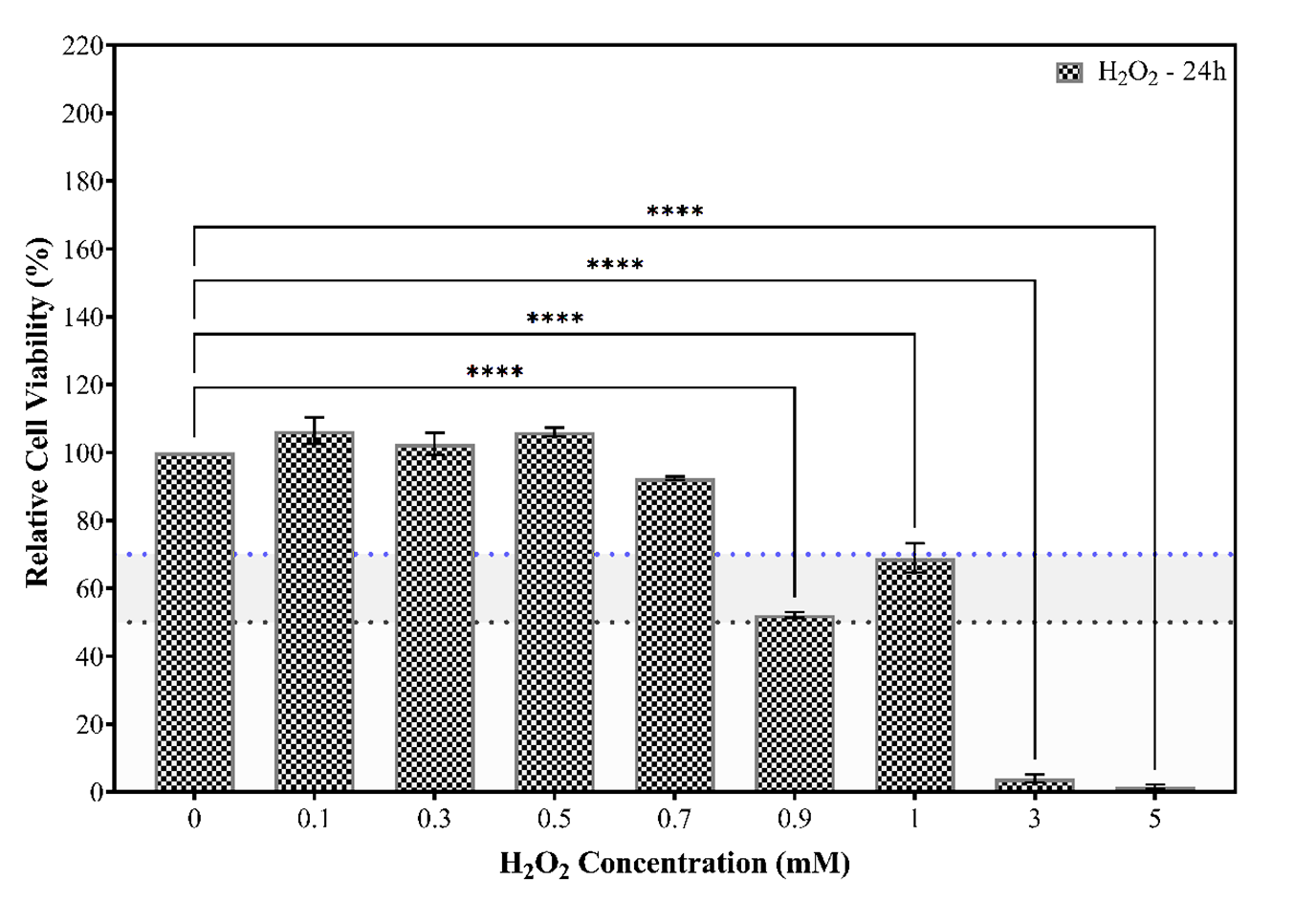


**Figure S5.** **Result from MTT assay.** The figure shows the cytotoxicity rendered by hydrogen peroxide concentrations over a period of 24 hours in RAW 264.7 macrophages. The results are expressed as mean ± SEM (n=3).


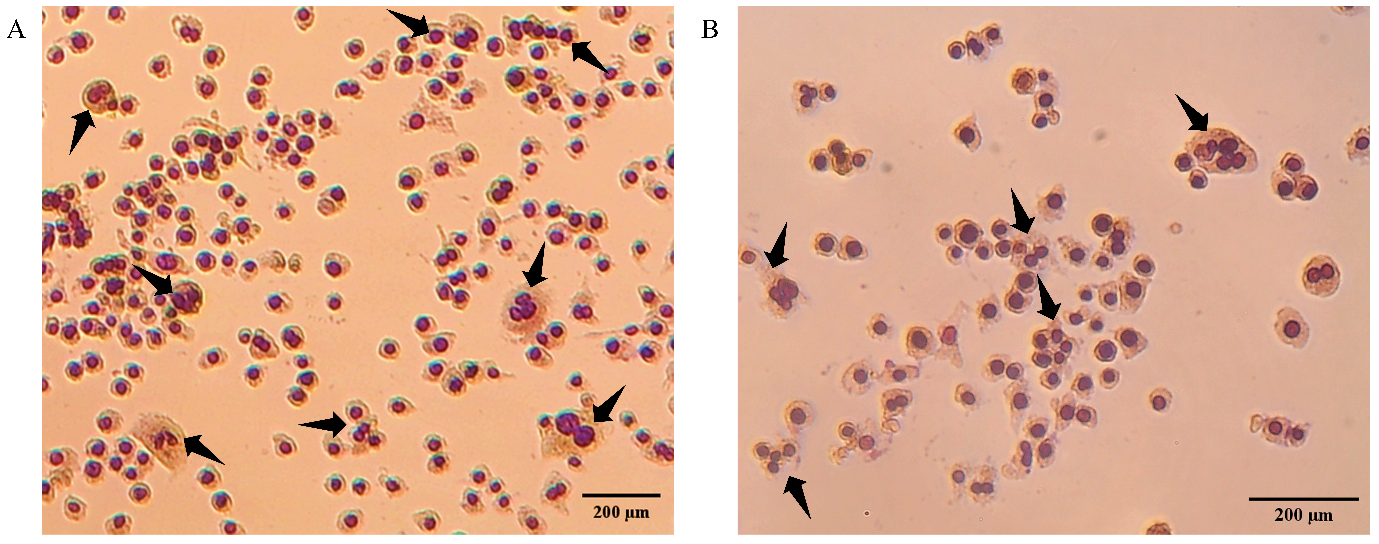


**Figure S6. The figure depicts osteoclast differentiation.** The panel **[A]** shows the differentiated osteoclasts at 4X magnification while the panel **[B]** shows the differentiated osteoclasts at the 10X magnification. The black arrows denote the differentiating osteoclasts with multiple nucleus at Day 5 of differentiation from mononucleated RAW 264.7 macrophages in the osteoclast differentiation media prepared for differentiation studies.

**Table S2.** The table shows the parameters involved in ADME screening using Swiss-ADME web server.

| ***Features*** | ***Taurine*** | ***TA-16*** | ***TA-39*** | ***TA-45*** | ***TA-47*** | ***TA-50*** | ***TA-52*** | ***TA-53*** | ***TA-64*** | ***TA-65*** | ***TA-70*** |
| --- | --- | --- | --- | --- | --- | --- | --- | --- | --- | --- | --- |
| ***TPSA*** | 88.77 | 151.52 | 114.79 | 174.76 | 137.53 | 151.52 | 134.12 | 74.78 | 108.92 | 131.29 | 127.57 |
| ***Consensus Log P*** | -1.43 | -2.14 | -2.07 | -2.41 | -1.42 | -2.14 | -1.12 | -0.52 | -0.41 | -1.35 | -1.27 |
| ***ESOL Log S*** | 2.11 | 2.27 | 2.5 | 2.05 | 0.58 | 2.27 | 0.03 | 0.85 | -1.17 | 1.35 | 1.62 |
| ***GI absorption*** | High | Low | High | Low | High | Low | High | High | High | High | High |
| ***BBB permeant*** | No | No | No | No | No | No | No | No | No | No | No |
| ***CYP1A2 inhibitor*** | No | No | No | No | No | No | No | No | No | No | No |
| ***CYP2C19 inhibitor*** | No | No | No | No | No | No | No | No | No | No | No |
| ***CYP2C9 inhibitor*** | No | No | No | No | No | No | No | No | No | No | No |
| ***CYP2D6 inhibitor*** | No | No | No | No | No | No | No | No | No | No | No |
| ***CYP3A4 inhibitor*** | No | No | No | No | No | No | No | No | No | No | No |
| ***log Kp (cm/s)*** | -9.98 | -11.14 | -10.63 | -10.92 | -9.16 | -11.14 | -8.32 | -10.14 | -7.01 | -10.35 | -9.86 |
| ***Lipinski #violations*** | 0 | 0 | 0 | 0 | 0 | 0 | 0 | 0 | 0 | 0 | 0 |
| ***PAINS #alerts*** | 0 | 0 | 0 | 0 | 0 | 0 | 0 | 0 | 0 | 0 | 0 |
| ***Synthetic Accessibility*** | 2.17 | 2.97 | 2.22 | 2.87 | 2.56 | 2.93 | 2.95 | 2.84 | 2.45 | 3.71 | 2.86 |

**Table S3.** The table shows the parameters involved in toxicity screening using ProTox-II web server.

| ***Analogues*** | ***Hepatotoxicity*** | ***Carcinogenicity*** | ***Immunotoxicity*** | ***Mutagenicity*** | ***Cytotoxicity*** |
| --- | --- | --- | --- | --- | --- |
| ***TA-39*** | 0.78 | 0.73 | 0.99 | 0.69 | 0.61 |
| ***TA-47*** | 0.89 | 0.63 | 0.99 | 0.67 | 0.57 |
| ***TA-52*** | 0.89 | 0.64 | 0.99 | 0.64 | 0.6 |
| ***TA-53*** | 0.88 | 0.68 | 0.99 | 0.65 | 0.56 |
| ***TA-64**** | **NA** | **NA** | **NA** | **NA** | **NA** |
| ***TA-65*** | 0.83 | 0.78 | 0.98 | 0.56 | 0.59 |
| ***TA-70*** | 0.77 | 0.8 | 0.99 | 0.69 | 0.59 |

******* *Excluded value*


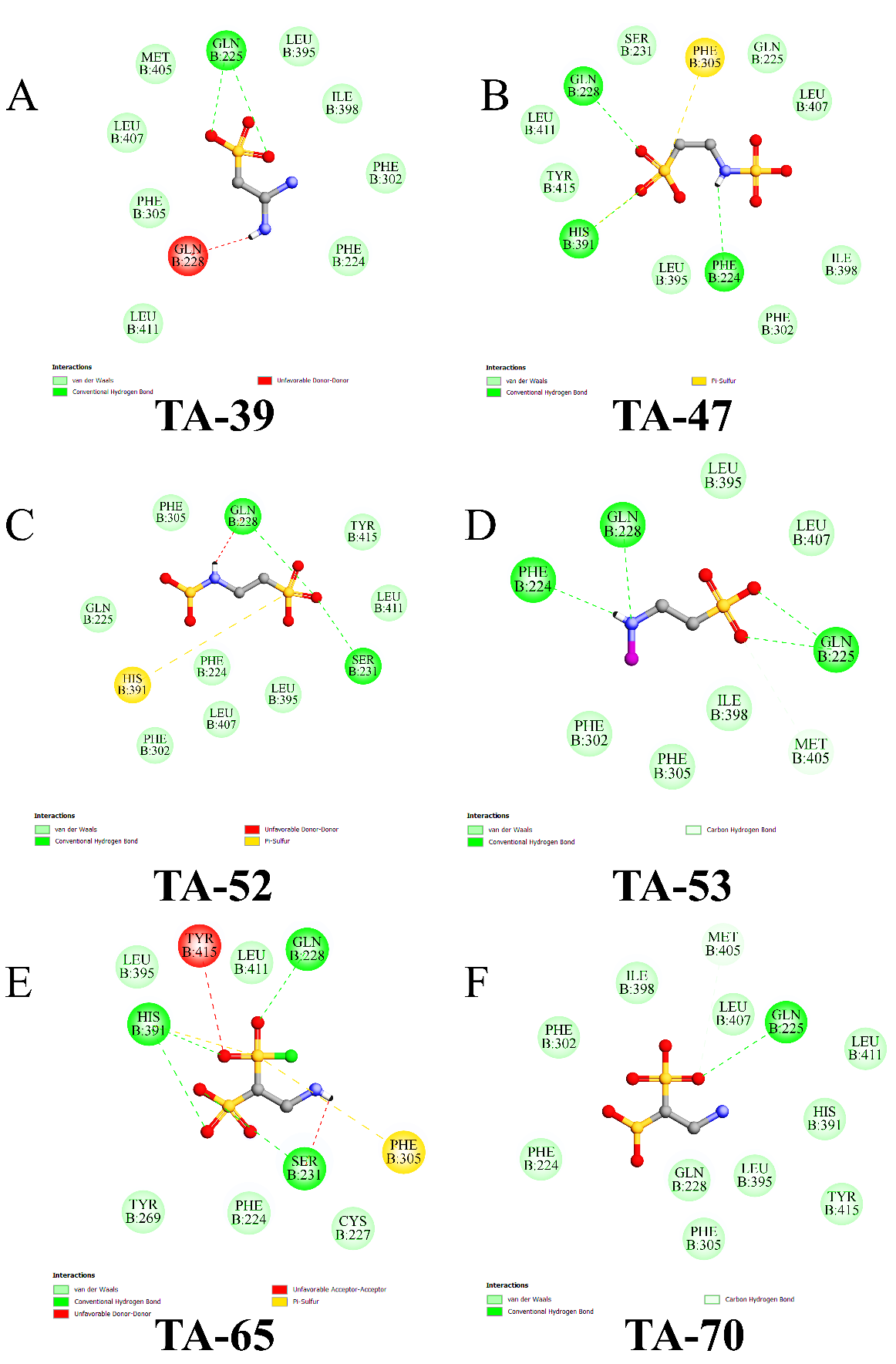


**Figure S7. Molecular docking 2-D interaction analysis for the final six taurine analogues.** The panel **[A]** is TA-39, **[B]** TA-47, **[C]** TA-52, **[D]** TA-53, **[E]** TA-65, and **[F]** TA-70
